# Diversity analysis of cotton (*Gossypium hirsutum* L.) germplasm using the CottonSNP63K Array

**DOI:** 10.1186/s12870-017-0981-y

**Published:** 2017-02-03

**Authors:** Lori L. Hinze, Amanda M. Hulse-Kemp, Iain W. Wilson, Qian-Hao Zhu, Danny J. Llewellyn, Jen M. Taylor, Andrew Spriggs, David D. Fang, Mauricio Ulloa, John J. Burke, Marc Giband, Jean-Marc Lacape, Allen Van Deynze, Joshua A. Udall, Jodi A. Scheffler, Steve Hague, Jonathan F. Wendel, Alan E. Pepper, James Frelichowski, Cindy T. Lawley, Don C. Jones, Richard G. Percy, David M. Stelly

**Affiliations:** 1 USDA-ARS, Crop Germplasm Research Unit, College Station, TX 77845 USA; 2 Department of Plant Sciences and Seed Biotechnology Center, University of California-Davis, Davis, CA 95616 USA; 3 CSIRO Agriculture & Food, Black Mountain Laboratories, Canberra, ACT 2601 Australia; 4 USDA-ARS, Cotton Fiber Bioscience Research Unit, New Orleans, LA 70124 USA; 5 USDA-ARS, Cropping Systems Research Laboratory, Plant Stress and Germplasm Development Research Unit, Lubbock, TX 79415 USA; 6 CIRAD, UMR AGAP, Montpellier, F34398 France; 7 EMBRAPA, Algodão, Nucleo Cerrado, 75.375-000 Santo Antônio de Goias, GO Brazil; 8 Plant and Wildlife Science Department, Brigham Young University, Provo, UT 84602 USA; 9 USDA-ARS, Jamie Whitten Delta States Research Center, Stoneville, MS 38776 USA; 10 Department of Soil & Crop Sciences, Texas A&M University, College Station, TX 77843 USA; 11 Department of Ecology, Evolution, and Organismal Biology, Iowa State University, Ames, IA 50011 USA; 12 Department of Biology, Texas A&M University, College Station, TX 77843 USA; 13 Interdisciplinary Department of Genetics, Texas A&M University, College Station, TX 77843 USA; 14 Illumina Inc., 499 Illinois Street, San Francisco, CA 94158 USA; 15 Cotton Incorporated, Agricultural Research, Cary, NC 27513 USA

**Keywords:** Breeding, Cotton, Diversity analysis, Genome-wide association analysis, Germplasm collection, Molecular markers, Seed protein content

## Abstract

**Background:**

Cotton germplasm resources contain beneficial alleles that can be exploited to develop germplasm adapted to emerging environmental and climate conditions. Accessions and lines have traditionally been characterized based on phenotypes, but phenotypic profiles are limited by the cost, time, and space required to make visual observations and measurements. With advances in molecular genetic methods, genotypic profiles are increasingly able to identify differences among accessions due to the larger number of genetic markers that can be measured. A combination of both methods would greatly enhance our ability to characterize germplasm resources. Recent efforts have culminated in the identification of sufficient SNP markers to establish high-throughput genotyping systems, such as the CottonSNP63K array, which enables a researcher to efficiently analyze large numbers of SNP markers and obtain highly repeatable results. In the current investigation, we have utilized the SNP array for analyzing genetic diversity primarily among cotton cultivars, making comparisons to SSR-based phylogenetic analyses, and identifying loci associated with seed nutritional traits.

**Results:**

The SNP markers distinctly separated *G. hirsutum* from other *Gossypium* species and distinguished the wild from cultivated types of *G. hirsutum*. The markers also efficiently discerned differences among cultivars, which was the primary goal when designing the CottonSNP63K array. Population structure within the genus compared favorably with previous results obtained using SSR markers, and an association study identified loci linked to factors that affect cottonseed protein content.

**Conclusions:**

Our results provide a large genome-wide variation data set for primarily cultivated cotton. Thousands of SNPs in representative cotton genotypes provide an opportunity to finely discriminate among cultivated cotton from around the world. The SNPs will be relevant as dense markers of genome variation for association mapping approaches aimed at correlating molecular polymorphisms with variation in phenotypic traits, as well as for molecular breeding approaches in cotton.

**Electronic supplementary material:**

The online version of this article (doi:10.1186/s12870-017-0981-y) contains supplementary material, which is available to authorized users.

## Background

Cotton (*Gossypium* spp.), primarily the tetraploid species, *Gossypium hirsutum* L., is produced in many diverse tropical, subtropical and warm temperate regions of the world. Because cotton cultivation is so extensive, *ca.* 31 million hectares worldwide in 2016 (USDA-NASS), the societal benefits from plant breeding-based gains in cotton performance are greatly magnified. These include improved natural resource conservation and preservation, reduced reliance on undesirable chemical protectants, more time- and energy-efficient production, and increased profits for growers and cotton-related businesses, as well as local and even national economies. Improvements in baseline crop production and product quality have been possible through breeding of cotton germplasm resources, especially genetically elite cultivars. The use of non-elite germplasm resources for genetic improvement has been increasing in part because molecular markers and sequence polymorphisms facilitate germplasm analysis, classification, categorization, genotyping, genomic comparisons, and various types of marker-assisted selection for breeding and breeding-related research.

The importance of cotton fiber as a commodity is widely recognized due to its extensive use in textiles. The importance of cottonseed as a source of cooking oil and feed for cattle is less recognized and has been far less studied. Renewed interest in cottonseed for extended use as feed for non-ruminants or even human consumption comes from the increasing need for affordable sources of protein to feed a growing global population [1] and the ability to selectively eliminate gossypol from the seed [2]. Increased demand for feed and food will likely change across time and increase seed usage. Regardless, global climate changes will require cultivars adapted to higher temperatures, decreased precipitation, and/or increased salinity along with new genotypes with resistance to altered profiles of pests and pathogens. To meet these challenges, plant breeders will need to identify novel sources of variation and incorporate them into their breeding programs.

Cotton germplasm resources available worldwide [3] contain beneficial allelic variations that traditional and genomic breeding methods can exploit to develop cultivars adapted to emerging environmental and climate conditions. The USDA National Cotton Germplasm Collection (NCGC) is one of the largest collections of cotton germplasm resources [3]. The NCGC is comprised of over 10,000 accessions representing nine genomes and 45 species originating worldwide. However, the unequivocal differentiation, categorization, and classification of accessions remain challenging for *Gossypium* germplasm resources [4]. In some cases, race and species designations remain ambiguous [5–9]. Continual characterization and evaluation using the latest technologies are essential to providing the most accurate information to potential germplasm users. The ‘*Gossypium* Diversity Reference Set’ (GDRS) is a subset of approximately 20% of the entire NCGC, created in 2013 via proportional representation across taxonomic levels and geographic origins [4]. Simple sequence repeat (SSR) markers were recently used to genotype the GDRS to evaluate the applicability of a core set of markers across multiple genera and species [4] and to identify differences between individual accessions and groups of wild and improved types of *G. hirsutum* and *G. barbadense* L. [10]. Based on this information, core sets of *G. hirsutum* and *G. barbadense* are being developed for future genetic studies. The GDRS has been evaluated for seed oil, protein, and seed index traits [11], and subsets are being evaluated for other agronomic traits.

Diverse resources within other worldwide cotton germplasm collections have also been characterized with molecular markers, primarily SSRs [7, 12–14]. Additionally, elite cultivars from breeding programs are often genotyped to enable molecular comparisons of cultivars, known pedigrees, and the respective breeding germplasm resources in specific countries and growing regions [15–20]. The accumulation of knowledge regarding genetic differences is especially useful when combined with knowledge of differences in phenotypes because it allows breeders and other researchers to generate segregating populations of increased usefulness by choosing parents with complementary phenotypic traits and genetic distinctiveness. These populations are then utilized to combine and select favorable alleles; map and determine the genetic basis of a particular phenotype; and launch development of advanced cultivars.

With decreasing resources available for germplasm preservation, optimization of these resources becomes essential. Unrecognized redundancy of germplasm within collections has played a large role in impeding optimal management and use of collections [21]. It has been estimated that only one-third of the total number of accessions in rice germplasm banks may be distinct [22], and this situation to greater or lesser extent is recognized in many germplasm collections. Management of any plant collection requires a multitude of resources to maintain a highly viable seed inventory to ensure seed is available for utilization from the collection at any time. For cotton, the photoperiod sensitivity of many lines requires costly field and nursery production in two or more latitudes. Thus, maintenance of duplicated materials leads to considerable amounts of unnecessary effort and cost. Another concern in the management of germplasm collections and breeding programs is the maintenance of purity. Purity of lines is compromised by outcrossing, much of which cannot be easily detected by phenotypic observations. Accurate genotypic profiles in addition to phenotypic profiles are increasingly relied upon in purity testing of commercial cultivars [23]. Genotypic profiling is a sought after tool for germplasm resource management and is relevant to germplasm collections of cotton, which is self-pollinated yet highly amenable to insect-mediated cross-pollination [24].

Most recently in cotton, SSRs have been the marker of choice for researchers to identify genotypic differences [10, 12, 13, 15, 17, 25]. SSR markers typically can detect many alleles per locus, resulting in a high polymorphism which allows for utilization of a smaller number of markers. However, genotyping with SSRs is time-consuming, moderately costly, and difficult to do in a high-throughput manner [26]. In contrast, single nucleotide polymorphisms (SNPs), only have two alleles at each locus, and therefore a single marker is less informative at a locus than an SSR. However, SNPs occur more frequently than SSRs throughout the genome, and greater numbers of markers can be obtained. The benefit of SNPs is that it is possible to genotype a large number of SNPs simultaneously in a high-throughput, cost effective manner due to their bi-allelism. When diversity analyses were conducted with an equal number of SSRs and SNPs in maize, the SSR analysis was generally more informative [26]. But when the number of SNPs was increased, this limitation was overcome, and SNPs were able to resolve differences between extremely similar individuals as well as increase the accuracy of diversity estimates [26, 27].

Compared to many field crops, cotton has only recently begun to benefit from the wide availability of SNP markers. Allotetraploid crops like cultivated cotton are especially challenging for the identification of SNPs [28, 29]. Most efforts on cotton SNPs to date have focused mainly on discovery and validation [30–37]. These efforts have culminated in the identification of sufficient SNP markers to establish high-throughput genotyping systems, such as the CottonSNP63K array. These systems enable researchers to efficiently analyze large numbers of SNP markers and obtain highly repeatable results [38]. In the current investigation, we aim to utilize this SNP array for analyzing diversity within a predominantly *G. hirsutum* cultivar set and to identify loci associated with seed nutritional traits.

To obtain a more thorough characterization of the genetic diversity of cotton, we used the CottonSNP63K array to analyze a total of 395 cotton samples provided by the NCGC and collaborators worldwide. The data collected on the samples shows how this technology can be applied 1) to evaluate genetic diversity and population structure between groups of germplasm, 2) to gauge the effectiveness of the array to identify differences among individual *G. hirsutum* genotypes, 3) to investigate possible redundancies in NCGC accessions, 4) to relate this new technology with results from SSR markers, and 5) to explore the potential of the array for use in genome-wide association studies.

## Methods

### Plant material and genotyping

Genomic DNA was extracted from young leaves of single plants representing a panel of 395 diverse *Gossypium* genotypes following the protocol described in Hulse-Kemp et al. [38]. DNA from young leaves was extracted using NucleoSpin Plant II kits (MACHEREY-NAGEL, USA), quantified using PicoGreen, and normalized to 50 ng/μL prior to genotyping. Five of these samples (all improved genotypes developed in cotton breeding regions outside the United States) were removed from further analysis due to potential misclassification or admixture (Additional file 1), leaving 390 genotypes for analysis. The final panel included 363 *G. hirsutum* samples; of which 292 were improved/cultivated or previously cultivated types and 71 were non-cultivated relatives (Additional file 2). The remaining 27 samples were from 10 diploid and tetraploid *Gossypium* species other than *G. hirsutum*. The non-*G. hirsutum* species included six diploid species (*G. arboreum*, *G. amourianum*, *G. longicalyx*, *G. raimondii*, *G. thurberi*, and *G. trilobum*) and four tetraploid species (*G. barbadense*, *G. ekmanianum*, *G. mustelinum*, and *G. tomentosum*). All plant materials used in this study were obtained from other researchers or national germplasm collections as indicated in Additional file 2.

The improved types of *G. hirsutum* were selected to span global regions of cotton breeding efforts. Within improved types, 185 cultivars were developed in the United States, and these were further classified into four breeding regions: eastern, mid-south, plains, and western. For a detailed description of criteria used for classification into global and US breeding regions see Hinze et al. [10]. Classification was based on declared passport data at time of submission. To provide continuity with our earlier report [10], genotypes from Africa were separated into two groups, northern Africa and southern Africa, with the equator as the boundary. We theorized that genetic differences observed between African improved germplasm would be based less upon geospatial considerations and more on breeding history. It was thought that genetic groupings might exist based on previous colonization and trade relations. The two regional groups were arbitrary but have been preserved for continuity. Within the improved group of *G. hirsutum*, 15 examples of genotypes with the same designations were included to examine whether genotypes with the same names were also genetically similar. In 12 examples, the genotypes came from different breeding programs (biological replicates), and in the remaining examples, the genotypes came from the same seed source (technical replicates) (Additional file 3).

Analyses of non-cultivated *G. hirsutum* germplasm centered around seven geographical landraces: *latifolium*, *marie-galante*, *morrilli*, *palmeri*, *punctatum*, *richmondi*, and *yucatanense* [5]. These analyses of non-cultivated types included a distinct group of “mocó” cotton (*G. hirsutum* race *marie-galante*), primarily of Brazilian origin [39]. For consistency with our earlier reports [4, 10, 11], the accessions from the non-cultivated perennial relatives of *G hirsutum*, i.e., from the seven geographical landraces, will be further and collectively referred to as “wild” even though it is recognized that the only truly wild landrace is *yucatanense*, while the remaining six landraces display some traces of domestication and are considered ferals, or relics from ancient pre-Columbian domesticated forms.

SNP genotypes were generated for each panel member according to Hulse-Kemp et al. [38], using the CottonSNP63K array (Illumina, USA), which contains 63,058 SNP Infinium II assays and the cluster file available for tetraploid *Gossypium* germplasm. The cluster file was developed using 1,156 cotton samples including the subset of plant materials used in this diversity analysis. Genotypes were determined for all samples in the present analysis using the 38,822 SNPs classified as polymorphic by Hulse-Kemp et al. [38]. Approximately 30% of the SNP assays in the CottonSNP63K were designed from 20,000 sequences for detecting polymorphism between *G. hirsutum* and five other species, namely *G. barbadense*, *G. tomentosum* Nuttal x Seemann, *G. mustelinum* Miers x Watt, *G. armourianum*, and *G. longicalyx*, but not among *G. hirsutum* genotypes.

### Data analysis

Standard summary statistics for all SNPs were generated using PLINK v. 1.90b3m (https://www.cog-genomics.org/plink2) [40, 41]. SNP allele frequencies were calculated using the ‘--freq’ option. Estimates of expected heterozygosity were calculated using the ‘--hardy’ option. Polymorphic SNPs were defined as those with a minor allele frequency (MAF) greater than 0.01within a defined set of samples. Unique SNPs were defined as those with MAF > 0 in a given group of samples and MAF = 0 in all other groups being compared. The number of homozygous differences between each line was calculated with the ‘bcftools gtcheck’ command in VCFtools [42].

Summary statistics and diversity analyses were independently evaluated on (1) the full dataset containing multiple *Gossypium* species, (2) a dataset of only *G. hirsutum* samples, (3) a dataset of only improved (i.e., cultivated) *G. hirsutum*, and (4) a dataset of only wild (i.e., landrace) *G. hirsutum*. The subset of improved *G. hirsutum* cultivars originating in US breeding programs was also evaluated.

Diversity was analyzed using SNPs with a MAF greater than 0.01 and a genotyping rate greater than 0.90. A genetic similarity matrix for all pair-wise combinations of individuals was calculated using PLINK (‘--cluster --matrix’ option) on the basis of the genome-wide average proportion of alleles shared which were identical by state (IBS) between any two individuals [41]. Multidimensional scaling (MDS) analysis of the genetic similarity matrix was used to extract the first six dimensions of relationships between cultivars with PLINK using the ‘--cluster --mds-plot 6’ option. Further interpretation of individual ancestry and degree of admixture was estimated using fastSTRUCTURE [43]. The three data sets (2–4), as noted above, were initially run for K = 1 to 10 with the ‘--prior = simple’ option and remaining default parameters. The optimal value of K for these runs was then determined using the ‘chooseK’ function. If the optimal K was determined to be 1, the process was repeated for K = 1 to 3 with the ‘--prior = logistic’ option. Distruct v. 1.1 as implemented in fastSTRUCTURE was used to generate bar plots to visualize proportions of admixture.

Putatively identical accessions from the NCGC, as determined by the matrix derived from SNP-based IBS values (IBS > 0.98) were compared for phenotypic similarity at the Southern Plains Agricultural Research Center (SPARC) in 2015. Seeds representing each accession were planted in the greenhouse in early April and grown for 3 weeks before transplanting to field plots. Field plots were 10.06 m × 1.02 m with 20 plants per plot to represent each accession. During the growing season, 26 morphological descriptors were scored as a plot average. This group of descriptors included leaf hair, leaf color, leaf shape, stem color, stem glands, stem hair, leaf size, leaf glands, leaf nectaries, bract nectaries, boll nectaries, petal color, pollen color, petal spot, stigma, bract type, bract teeth size, bract teeth number, bract color, boll shape, boll point, boll size, boll color, boll glanding, boll pitting, and fruiting type (for description of these traits, rating scale, and associated digital images, see https://www.cottongen.org/data/trait/NCGC_rating_scale). These descriptors are primarily inherited as qualitative traits whose expression shows negligible environmental interaction; therefore, characters were scored in a single replicate at a single location.

Among the 395 genotypes of the current investigation, a subset of 195 *G. hirsutum* accessions (126 improved and 69 wild types) were selected from the GDRS that were previously genotyped with SSR markers [4, 10] (Additional file 2). Genotype data of 105 SSR markers were obtained from the Dryad Digital Repository [44] and compared to data from 38,822 SNPs in the current analysis. Three of the improved *G. hirsutum* accessions were found to be admixtures based on SSR analyses and were removed prior to comparison of SNP and SSR results (Additional file 1). Independent analyses were conducted for the data subsets of a) 192 *G. hirsutum* accessions and b) 123 improved *G. hirsutum* accessions. Loci that were monomorphic within the respective subset were removed prior to calculation of genetic similarities. A genetic similarity matrix was calculated for each marker system based on Jaccard’s coefficient [45] as implemented in NT-SYS [46]. Genetic similarity values were plotted against each other and were compared with Mantel statistics via the ‘MxComp’ option in NT-SYS with 5,000 iterations to provide an α = 0.01 level of significance. Principal coordinate analyses (PCoA) were then calculated from Jaccard’s similarity values. To analyze SNP data using NT-SYS software, a text file set was generated from the PLINK binary file format using the ‘--recode 01’ option to code the minor alleles as ‘0’ and the major alleles as ‘1’, and the ‘--output-missing-genotype 9’ option to code missing data as ‘9’.

Phenotypic data for seed traits including percent oil content, percent protein content and seed index (grams per 100 seeds) were obtained from Hinze et al. [11] for the 195 accessions common to the previous SSR and current SNP study. The trait data was measured on seeds available in the NCGC which were harvested across different locations and years. The SNP genotypes for these lines were categorized and filtered for MAF greater than 0.01 and a genotyping rate greater than 0.90. Binary PED files for genotypes and phenotypes were exported from PLINK and used as input for single-SNP based association testing using the Genome-wide Efficient Mixed Model Association software (GEMMA) [47]. GEMMA was first applied to calculate new relationship matrices that could be used to adjust for population structure for each trait. QTL associations were then analyzed using the univariate linear mixed model equation. Three statistical tests were calculated, including the Wald test, likelihood ratio test, and the score test. Correction for population structure was assessed by plotting the observed versus expected ratios for each phenotype. Obtained *p*-values were filtered for significance using the Benjamini-Hochberg’s False Discovery Rate (FDR) method [48]. The FDR was initially constrained to the level α = 0.05, but values up to 0.15 were used when few results were obtained using the initial α level. Candidate genes located near the determined loci were investigated using alignment information of SNPs to the *G. raimondii* (D_5_) reference genome and/or the NBI (Novogene Bioinformatics Institute) *G. hirsutum* (TM-1, [AD]_1_) draft genome [49, 50].

## Results

### Minor allele frequencies within and across *Gossypium* germplasm groups

We used a high-throughput genotyping platform to appraise the diversity of cultivated cotton and its wild relatives (Additional file 2). Of the SNPs identified in this study, 33,507 (86%) were polymorphic across all *Gossypium* species measured (Table 1). From the 20,000 sequences specifically selected to detect polymorphism between *G. hirsutum* and five other species, the array enabled detection of 17,954 interspecific polymorphisms (interspecific SNPs), which are useful for introgression research and breeding.

**Table 1:** Measures of SNP-based genetic diversity within *G. hirsutum* germplasm groups

		Average MAF			
Group	N	Including monomorphic	Excluding monomorphic	Total polymorphic SNPs (proportion)	Genetic diversity (H_E_)
*Gossypium* spp.	390	0.184	0.213	33507 (0.86)	0.249
*G. hirsutum*	363	0.169	0.252	25829 (0.67)	0.225
*G. hirsutum*, improved	292	0.145	0.242	23145 (0.60)	0.195
*G. hirsutum*, wild	71	0.177	0.260	26299 (0.68)	0.232
*G. hirsutum*, improved, US	185	0.141	0.241	22626 (0.58)	0.190
*G. hirsutum*, improved, other countries	107	0.147	0.248	22961 (0.59)	0.197
*G. hirsutum*, improved, US, eastern	48	0.132	0.234	21810 (0.56)	0.177
*G. hirsutum*, improved, US, mid-south	48	0.111	0.222	19357 (0.50)	0.151
*G. hirsutum*, improved, US, plains	43	0.135	0.245	21350 (0.55)	0.183
*G. hirsutum*, improved, US, western	12	0.118	0.267	17093 (0.44)	0.157
*G. hirsutum*, improved, US, n/a	34	0.142	0.231	23851 (0.62)	0.193

Number of polymorphic SNPs (MAF ≥ 0.01) was calculated out of 38,822 SNPs. *N* sample size, *MAF* minor allele frequency

Within *G. hirsutum*, the number of polymorphic SNPs (MAF > 0.01) ranged from 26,299 (68%) within 71 non-cultivated landraces to 23,145 (60%) in 292 cultivars; 25,829 (67%) SNPs were polymorphic within this set of *G. hirsutum*. The distribution of MAF across these groups indicated that improved cultivars, and more specifically, those from the western US breeding region, had the most monomorphic SNPs (40 and 56%, respectively) (Additional file 4). The utility of the SNP array was examined by characterizing SNP allele frequencies across the panel, revealing that nearly 86% of the called SNPs were polymorphic among the 390 cotton samples, with an average minor allele frequency of 0.21 across the entire set. The average MAF when determined for *G. hirsutum* alone was 0.25, considerably higher than over all *Gossypium* species tested. These results serve as a reminder that the array was designed for genotyping and discriminating among genotypes of *G. hirsutum*, their hybrids, and interspecific introgression products [38]. Based on MAF, for an average pairwise combination of any two improved types, ca. 5,650 SNPs would be expected to be detected, and for an average combination of one improved and one wild *G. hirsutum*, ca. 6,892 SNPs would be detected (Table 2). The observed number of homozygous SNP differences for the specific cultivars in this study averaged 7,017 SNPs with a maximum of 11,759 SNPs between ‘TAMCOT Sphinx’ and ‘Sealand2’ (an interspecific cross between *G. barbadense* and *G. hirsutum*) (Additional file 5). For the twelve groups of genotypes with the same names but from different seed sources, we observed a maximum of 4,857 homozygous differences between sources of the ‘PD 1’ cultivar (Additional file 3).

**Table 2:** Average expected number of SNPs between a pair of genotypes based on *Gossypium* germplasm group

		*G. hirsutum*			*G. hirsutum*, improved		*G. hirsutum*, improved, US				
Group	*Gossypium* spp.	Overall	Improved	Wild	US	Other countries	Eastern	Mid-south	Plains	Western	N/A
*Gossypium* spp.	7167.8										
*G. hirsutum* overall	6866.0	6564.2									
Improved	6408.7	6107.0	5649.7								
Wild	7029.7	6727.9	6270.6	6891.5							
*G. hirsutum*, improved											
US	6334.5	6032.7	5575.5	6196.4	5501.2						
Other countries	6441.7	6139.9	5682.7	6303.6	5608.5	5715.7					
*G. hirsutum*, improved, US											
Eastern	6154.2	5852.5	5395.2	6016.1	5321.0	5428.2	5140.7				
Mid-south	5744.1	5442.3	4985.1	5606.0	4910.8	5018.1	4730.6	4320.4			
Plains	6212.6	5910.9	5453.6	6074.5	5379.4	5486.6	5199.1	4789.0	5257.5		
Western	5875.9	5574.1	5116.9	5737.8	5042.6	5149.8	4862.4	4452.2	4920.8	4584.0	
N/A	6350.6	6048.8	5591.5	6212.5	5517.3	5624.5	5337.0	4926.9	5395.5	5058.7	5533.4

Calculations were based on the average MAF (including monomorphic SNPs) for each group obtained from Table 1

**Fig. 1:**
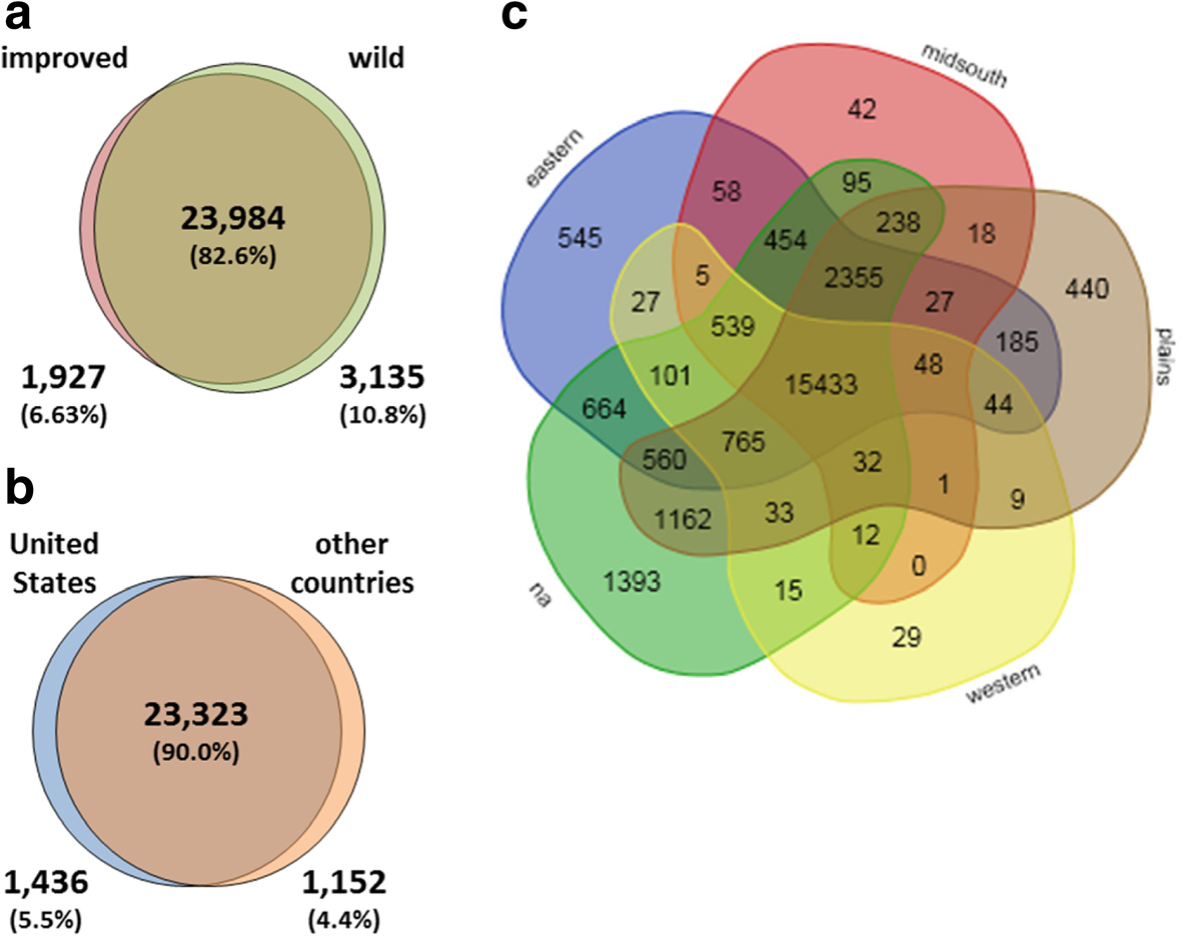
SNPs unique and common to different sets of *G. hirsutum* germplasm. **a** 292 improved and 71 wild samples, **b** improved samples from the United States (185) and from other countries (107), and **c** improved types from breeding regions within the United States (eastern, 48 samples; mid-south, 48; plains, 43; western, 12; n/a (unclassified breeding region), 34)

SNPs were initially characterized by their unique occurrence in several groups of *G. hirsutum*. In comparisons of non-improved and improved types, a large proportion of SNPs (23,984; 82.6%) were common to both groups, indicating that most genetic variation from wild types was also found in improved *G. hirsutum*. Whereas 3,135 or 10.8% of the total SNPs found in wild *G. hirsutum* were unique, only 1,927 or 6.6% of the SNPs observed in improved *G. hirsutum* were unique (Fig. 1a). Among global breeding regions for improved *G. hirsutum*, the United States had the highest number of unique SNPs (1,436), followed by the 18 Australian samples (149) and the 13 samples from northern Africa (118) (Additional file 1). Due to unequal sampling sizes among individual global breeding regions, a more appropriate comparison might be between improved accessions of the United States (*N* = 185) and all other countries (*N* = 107). A large proportion of SNPs (23,323; 90.0%) (Fig. 1b) were shared between the US and other countries, reflecting the common origins of cultivars and the continuing exchange of germplasm. Within improved *G. hirsutum*, approximately the same number of unique SNPs was found within the US (1,436) as compared to all other global breeding regions combined (1,152).

Within US improved germplasm, cultivars that were not assigned to a regional breeding group (unclassified, “n/a”) possessed almost three times as many unique SNPs (1,393) as cultivars assigned to specific breeding regions (Fig. 1c). The “n/a” group was very diverse and included a wide range of genotypes. Cultivars from the eastern (545) and plains regions (440) had notably higher numbers of unique SNPs when compared to the mid-south (42) and western region (29). The eastern, mid-south, plains, and unclassified regions of the United States were represented by approximately the same number of samples (48, 48, 43, and 34, respectively) while the western region had the least representation (12). Therefore, the few unique SNPs from the western region could correspond to its low representation within the improved germplasm of the United States. The low number of unique SNPs in relation to the high representation of samples from the mid-south could be indicative of the development and perpetuation of a genotype that is highly adapted and successful to that region and that has adaptive capabilities in other breeding regions.

Genetic diversity was greater in wild germplasm of *G. hirsutum* (H_E_ = 0.232) than in improved germplasm (H_E_ = 0.195) of the species (Table 1). For cotton bred within the US, the greatest genetic diversity (H_E_ = 0.193) was found in the group of improved samples that could not be assigned to one of the four breeding regions. This supports the high number of unique SNPs within this group and suggests the presence of a distinctive combination of SNPs within a very diverse group of germplasm. The lowest diversity was observed in the mid-south (H_E_ = 0.151) which corresponds to the observed low number of unique SNPs.

### Genetic similarity and relationships between *Gossypium* breeding groups

**Fig. 2:**
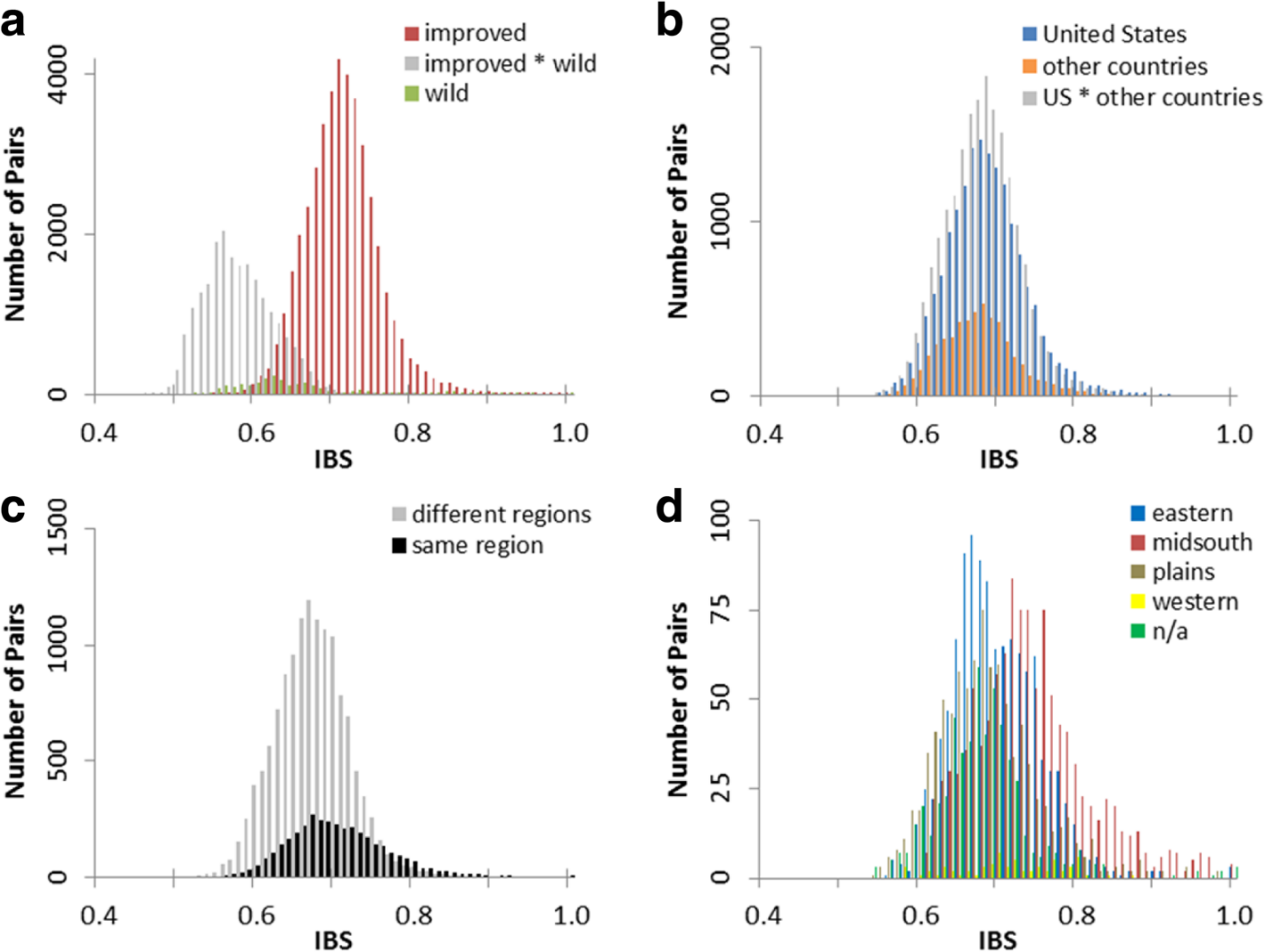
Identical by state (IBS) distributions for all pairwise sets of *G. hirsutum.* **a** improved versus wild samples, and improved samples from **b** US versus other countries, **c** between and within US regions overall, and **d** within US regions individually

The identical by state (IBS) values between pairs of individuals was used to estimate average genetic similarity within specific breeding groups. For the two groups of *G. hirsutum*, the highest similarity was observed in cultivars (IBS = 0.709) and the lowest similarity occurred among wild types (IBS = 0.652) (Table 3). Pairs of cultivars from the United States were only slightly more similar (IBS = 0.683) than pairs from other countries (IBS = 0.671). Within US breeding regions, the most similarity was observed among cultivars from the mid-south (IBS = 0.739) while the most dissimilar cultivars originated within the plains region (IBS = 0.682). Pairs of unclassified US cultivars were also highly dissimilar (IBS = 0.673), as expected among the assorted genotypes in this group. When comparing across US breeding regions, cultivars from the western and mid-south regions were the most different (IBS = 0.657). These pairwise genetic similarities may also be evaluated by their respective distribution patterns (Fig. 2a, b, c and d). Of particular interest was the relationship between the United States and other countries. The almost complete overlap in the distribution of pairwise similarity (or diversity) between improved cultivars worldwide was readily observed (Fig. 2b). Within the United States, increased genetic distance was more often found between pairs of cultivars from different regions than from the same region (Fig. 2c). This increased distance could be explained by the divergent distributions for IBS between regions (Fig. 2d). Within the wild category, some pairs had high similarity equivalent to or greater than that of improved pairs (Fig. 2a). Approximately 30% of the pairwise comparisons (representing 30 different wild samples) had IBS greater than 0.7. Upon further inspection, the majority of these samples in the pairwise comparisons had no race designation with the remainder being four *marie-galante*, four mocó, one *latifolium*, and one *morrilli* type. Based on geography of origin, several of the samples were from Venezuela, Brazil, Puerto Rico, and the Guadeloupe and Martinique Islands. This increased similarity could be indicative of a founder effect among the wild samples [51] where diversity was lost as the species moved from the Mexico and Guatemala center of diversity outward for domestication in the Caribbean and South America.

**Table 3:** Average proportion of alleles shared identical by state (IBS) as an estimate of genetic similarity

a)		Improved	Wild			
	Improved	0.709				
	Wild	0.573	0.652			
b)		US	Other countries			
	US	0.683				
	Other countries	0.671	0.671			
c)		Eastern	Mid-south	Plains	Western	N/A
	Eastern	0.694				
	Mid-south	0.680	0.739			
	Plains	0.659	0.665	0.682		
	Western	0.666	0.657	0.661	0.711	
	N/A	0.666	0.689	0.660	0.666	0.673

IBS values are calculated for groups based on a) 363 *G. hirsutum* samples, b) 292 improved *G. hirsutum* samples with global distribution, and c) 185 improved *G. hirsutum* samples from breeding regions within the United States

**Fig. 3:**
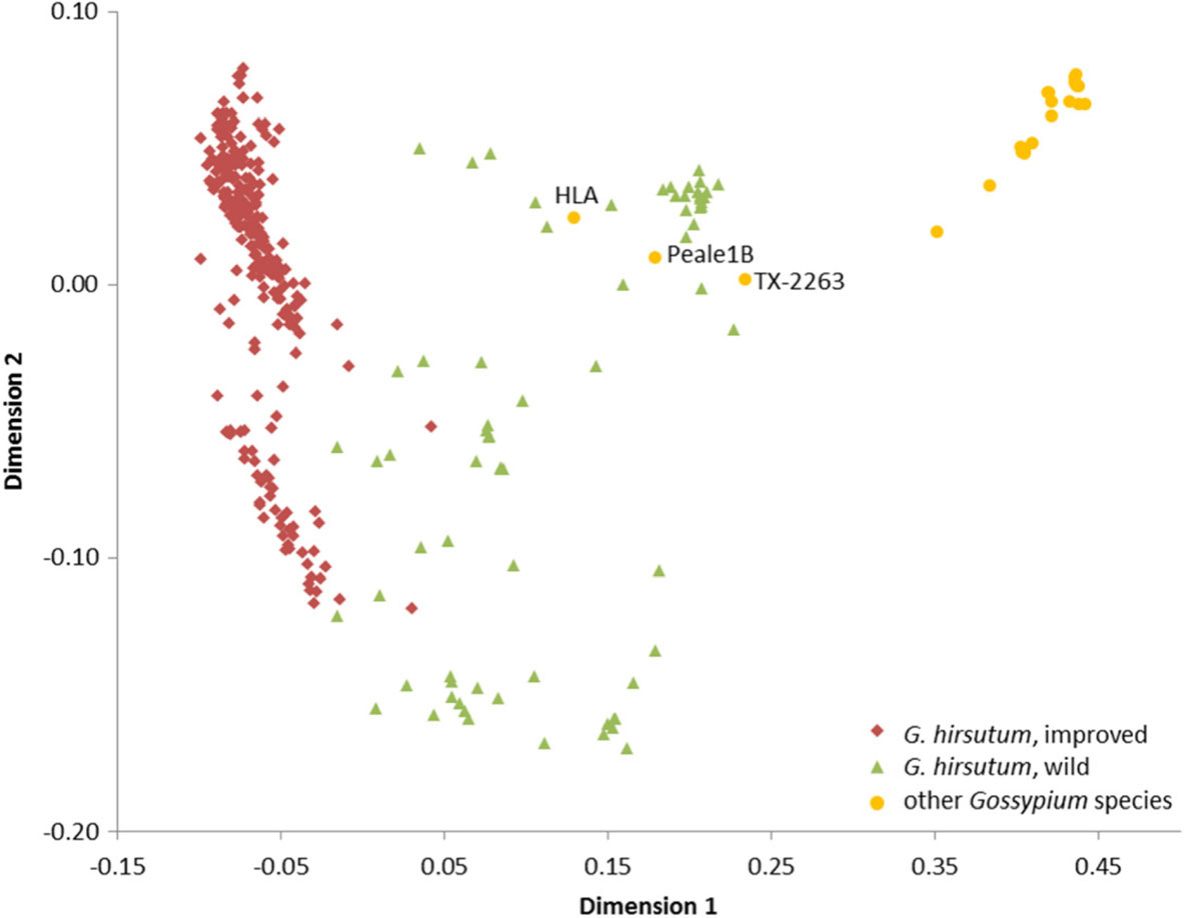
Two dimensional multidimensional scaling (MDS) plot of all *Gossypium* samples, showing separation of improved and wild (i.e. non-cultivated) forms of *G. hirsutum* from other *Gossypium* species. Identical by state genetic similarities of 390 *Gossypium* samples were used in generating the MDS plot. The three labelled samples are other *Gossypium* species that plotted similarly to wild samples of *G. hirsutum*

**Fig. 4:**
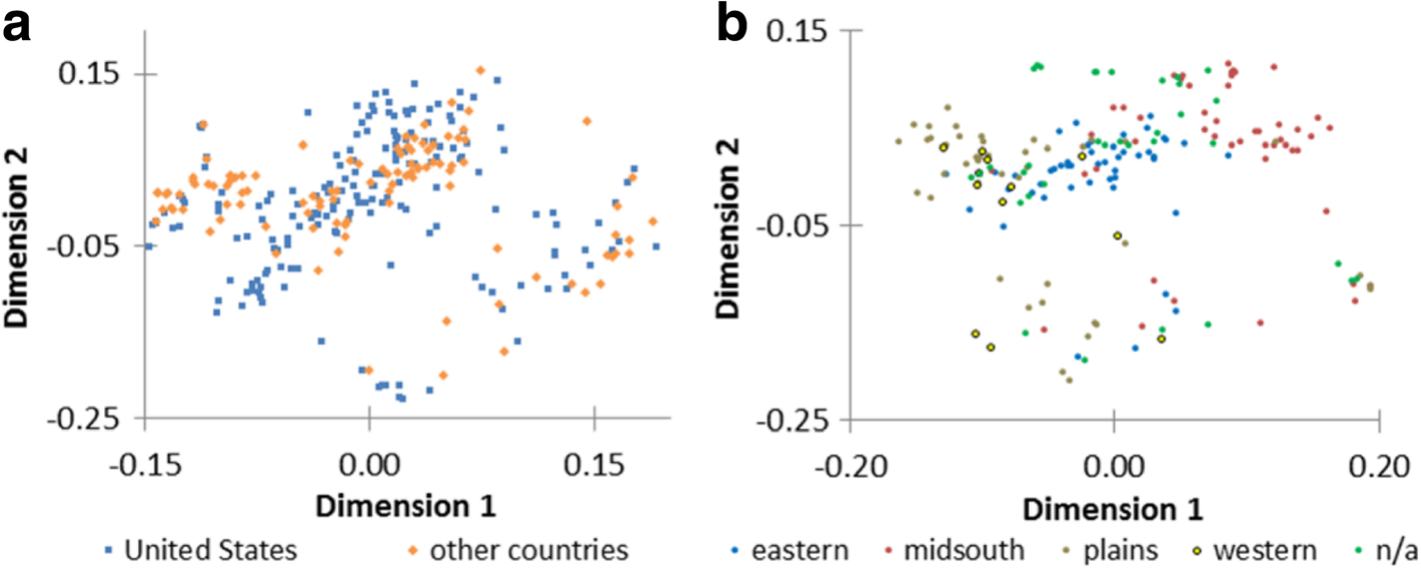
Two dimensional multidimensional scaling (MDS) plots of *G. hirsutum* groups. **a** improved type *G. hirsutum* samples from the United States and from other countries, and **b** improved type *G. hirsutum* samples from breeding regions in the United States. Identical by state genetic similarities of 185 *G. hirsutum* samples from the United States (eastern, 48; mid-south, 48; plains, 43; western, 12; n/a, 34) and 107 samples from other countries were used in generating the MDS plots

To examine genetic population structure and relationships among the major groups of germplasm, we conducted two independent tests of population stratification (MDS and fastSTRUCTURE). Multidimensional scaling (MDS) of pair-wise IBS values was used to visualize the relationships among several groups. First, all *Gossypium* species were analyzed together, and the SNPs efficiently separated *G. hirsutum* from all other species along the horizontal axis (Fig. 3). Three other *Gossypium* samples (not pure *G. hirsutum* lines) were noted at the edge of the range of wild samples of *G. hirsutum.* These samples included a synthetic tetraploid of *G. hirsutum* x *G. longicalyx* J.B. Hutchinson x *G. armourianum* Kearney (HLA); Peale1B, an unnamed new species from Wake Atoll, formerly *G. hirsutum* [52]; and TX-2263, recently confirmed as *G. ekmanianum* Wittmack [8] rather than a wild type of *G. hirsutum*. Within *G. hirsutum*, the wild and improved samples tended to separate along the horizontal axis with greater dispersion noted within the wild group. The improved cultivars generally separated into two clusters along the vertical axis. We were unable to determine a specific cause for this separation; however, it seems that it may be due to background in cultivars coming from the *G. hirsutum* genotype ‘TM-1’ [53]. This genotype is widely recognized as the genetic standard for *G. hirsutum* and thus has been included in a majority of genetic studies, including those for SNP discovery. Independent analysis of cultivars from the United States and other countries revealed a general mixing and overlapping of these two groups (Fig. 4a). Analysis of the 185 samples from United States breeding programs showed a tendency to stratify by breeding region along the horizontal axis; however, no distinct clusters were observed (Fig. 4b).

**Fig. 5:**
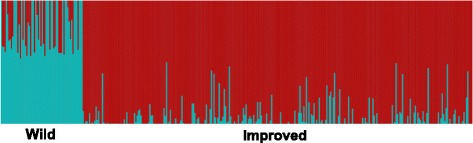
Estimated population structure for 363 *G. hirsutum* samples. fastSTRUCTURE bar plots for *K* = 2 show a clear split between improved and wild (i.e., non-cultivated landrace) samples

Further analyses of *G. hirsutum* diversity structure were run using fastSTRUCTURE software for three data sets: a) all *G. hirsutum*, b) only improved, and c) only wild or landrace samples. Only one of these analyses was able to determine a value of *K* that significantly clustered the data into more than one group. This occurred for the wild group where *K* = 7 was found to be significant, however there did not appear to be a known classification of samples to explain the observed clusters. The broad clustering of the improved samples into two clusters (indicated by MDS) was not detected by fastSTRUCTURE. The groupings suggested at higher *K* values did not correspond to any prior classifications and was generally consistent with the homogeneity of variation observed between countries in the MDS analyses. While it was not significant in explaining the genetic structure, the split between wild and improved groups was obvious at *K* = 2, in an analysis of all *G. hirsutum* (Fig. 5). Most wild samples did show evidence of varying levels of admixture with cultivated material, as expected since all cultivated material was fundamentally derived from wild germplasm.

### Phenotypic evaluation of genetically identical lines

In phenotypic comparisons of accession pairs determined to be highly similar (IBS > 0.98), only 7 of 26 morphological descriptors were found to differ within pairs. The descriptor ratings for leaf and boll nectary distributions differed most frequently among these paired accessions, followed closely by differences in ratings for quantity of stem hair (Table 4). One pair of accessions (‘MD51ne’ and ‘Siokra 104–90’) differed for five descriptors even though this pair had perfect genetic similarity (IBS = 1.000). There was no common breeding history to support identical genotypes between these two accessions. The plants used for genotyping and phenotyping grew from seeds obtained from the NCGC, but different plants were used for each evaluation; hence, there was opportunity for a mistake in sampling and/or handling that inadvertently may have led to these observations. Six of the remaining seven pairs differed in one to three descriptors. Only one pair (‘Deltapine 66’ and ‘Paymaster 54’) did not have phenotypic differences as indicated by descriptors. Among the highly similar accession pairs, this pair had one of the lowest tested pairwise IBS values (IBS = 0.981).

**Table 4:** Phenotypic descriptor values for eight pairs of accessions with high identical by state (IBS > 0.98) similarity

					Descriptors						
Pair	PI number	Designation	Pedigree Notes	IBS	Stem hair	Leaf hair	Leaf shape	Leaf nectaries	Bract nectaries	Boll nectaries	Boll shape
1	566941	MD51ne	DP90*3/MD65-11ne; MD65-11ne = FTA 263–2/4*DP 16 //2*Deltapine16ne; Deltapine 16ne = nectariless isoline of DP16	1.0000	moderate	moderate	**normal**	**absent**	**absent**	**absent**	**oval**
	607166	Siokra 104–90			moderate	moderate	**super okra**	**one, main vein**	**present**	**reduced**	**round**
2	529067	DES 716		0.9999	none	few	normal	**three**	present	**present**	round
	528649	Rowden	Sel. Bohemian		none	few	normal	**two**	present	**reduced**	round
3	528649	Rowden	Sel. Bohemian	0.9880	none	few	normal	one, main vein	present	**reduced**	round
	528634	Kekchi			none	few	normal	one, main vein	present	**present**	round
4	528634	Kekchi		0.9860	none	few	normal	**one, main vein**	present	present	round
	529067	DES 716			none	few	normal	**three**	present	present	round
5	529215	Auburn 56	Cook 37–6/2*CKR 1//CKR 1 W	0.9850	**moderate**	moderate	normal	**one, main vein**	present	present	round
	528655	Delfos 9169			**hairy**	moderate	normal	**three**	present	present	round
6	529565	Deltapine 66	DP16/DP554; DP16 = DP Smoothleaf/Fox 4–425; Fox 4–425 = DP 45 = Sel. Fox 4; DP554 = Auburn 56/DP15	0.9810	hairy	moderate	normal	three	present	reduced	round
	528820	Paymaster 54	Sel. Kekchi		hairy	moderate	normal	three	present	reduced	round
7	528970	Deltapine 14	DP 11/DP 1	0.9800	**moderate**	**moderate**	normal	one, main vein	present	**present**	round
	528649	Rowden	Sel. Bohemian		**none**	**few**	normal	one, main vein	present	**reduced**	round
8	529067	DES 716		0.9800	**none**	**few**	normal	one, main vein	present	present	round
	528970	Deltapine 14	DP 11/DP 1		**moderate**	**moderate**	normal	one, main vein	present	present	round

Seven of 26 descriptors showed differences between accessions when grown in the field at College Station, TX in 2015. Differences are highlighted in bold type

### Comparison between SNP and SSR markers in cotton

**Fig. 6:**
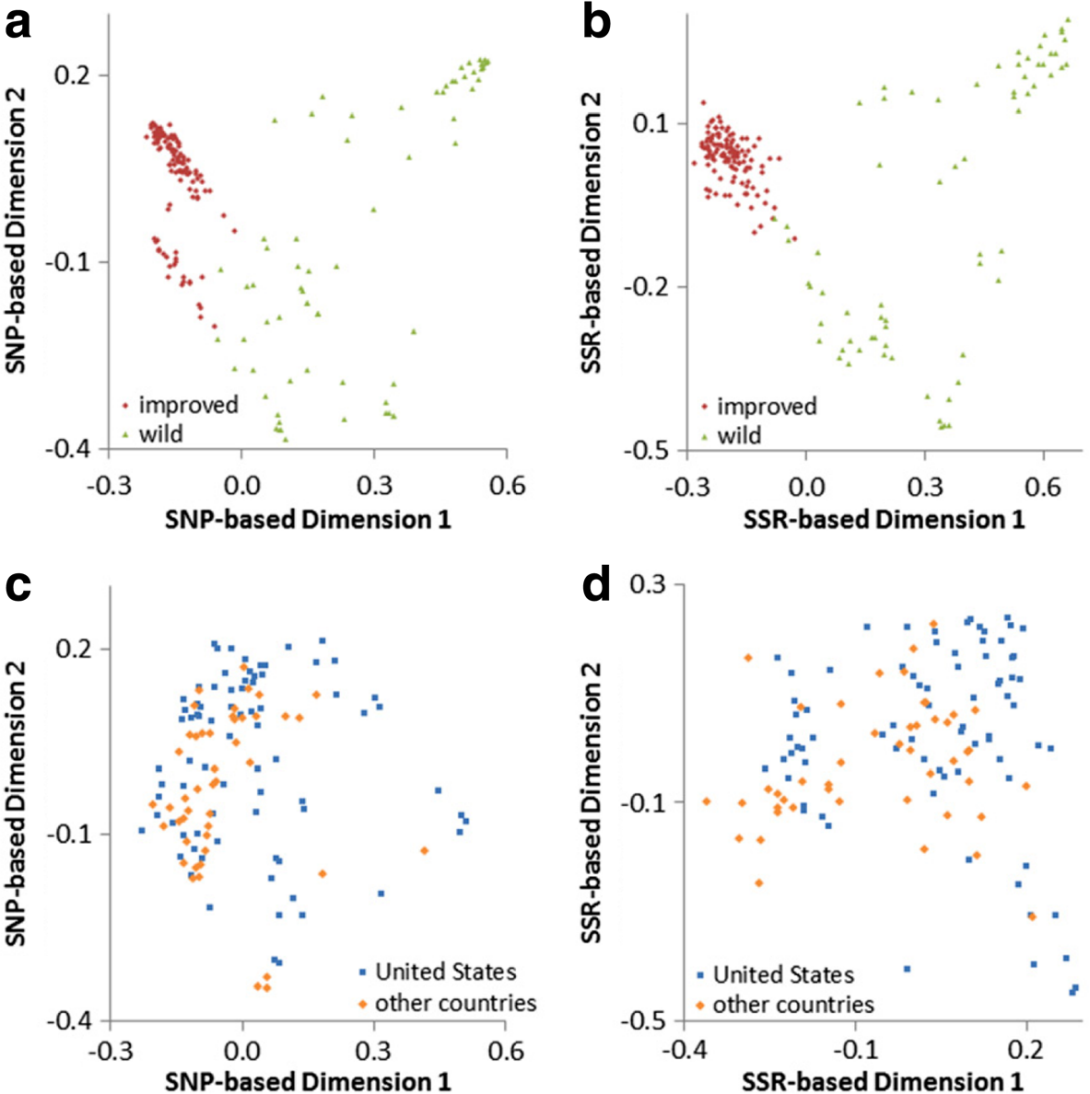
Comparisons of SNP and SSR principal coordinate analyses based on Jaccard’s coefficient. 192 *G. hirsutum* samples (123 improved and 69 wild types) from the US National Cotton Germplasm Collection were compared based on (**a**) 38,682 SNP loci and (**b**) 105 SSR loci. The 123 improved *G. hirsutum* samples (77 from the United States and 46 from other countries) were further independently analyzed using (**c**) SNP and (**d**) SSR loci

**Fig. 7:**
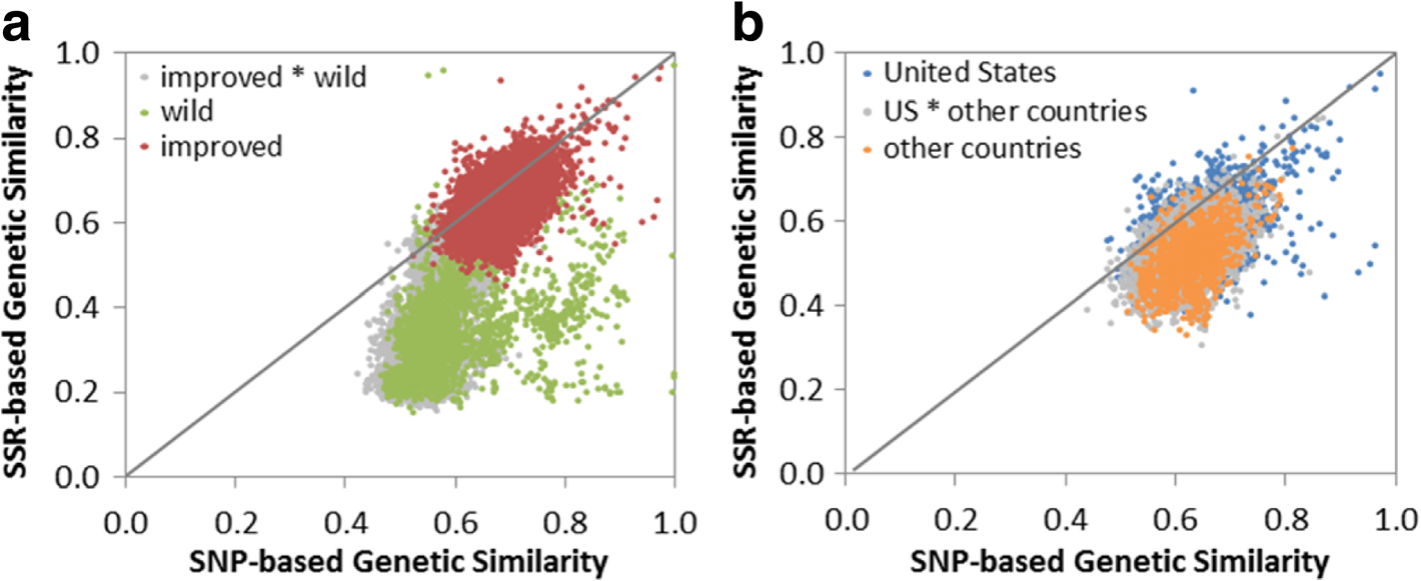
Relationship between SNP (x-axis) and SSR (y-axis) marker sets as calculated using Jaccard’s genetic similarity. **a** 192 *G. hirsutum* improved and wild samples (Mantel *r* = 0.798) and (**b**) 123 improved *G. hirsutum* samples grouped by global breeding region (Mantel *r* = 0.509). Each dot represents a pairwise comparison between samples

The planned commonality of 192 accessions between the current SNP investigation and a previous SSR investigation of diversity allows for defined comparisons to be made. Within this dataset, 26,324 SNPs and 103 SSRs (representing 748 alleles) were polymorphic. A key difference between the two studies is that a DNA bulk of 10 plants was used to obtain SSR data from individual accessions, whereas DNA from a single individual was used in the current study to obtain SNP data. One expected difference due to sampling technique was that SSR data should reveal larger genetic distances among heterogeneous accessions (generally the wild accessions) than one would expect to see in the improved accessions, which are more homogeneous. We compared the genetic relationships among these accessions for the two marker types using NTSYS. The PCoA plots revealed an obvious separation of improved from wild accessions along the horizontal axis for both marker systems (Fig. 6a and b). The relationship of improved *G. hirsutum* accessions from the United States and other countries was non-discriminatory for both marker types (Fig. 6c and d). When SNP and SSR data were combined (data not shown), the distribution of points in the PCoA plots closely resembled the distribution obtained when using solely SNP data. The Mantel *r* statistic of 0.798 indicated that there was relatively strong positive correlation between the SNP and SSR Jaccard similarity matrices for the *G. hirsutum* accessions (Fig. 7a). When comparing the matrices for only the improved accessions, the correlation was slightly lower, with a Mantel *r* statistic of 0.509 (Fig. 7b). Therefore, similarities among improved accessions calculated using SNPs were positively, but weakly related to similarity based on SSRs.

The array structure affected the calculated relationships among wild and cultivated accessions observed in the SNP marker system in a manner different from the SSRs. In Fig. 7a, the wild accessions (green) were skewed towards higher values based on SNP genetic similarity on the x-axis compared to the y-axis for the SSRs. This effect is likely due to the fact that a large number of markers were included on the array to produce an acceptable number of polymorphisms between any two improved *G. hirsutum* accessions. While a relatively smaller number of SNPs derived from wild accessions were also included on the array, thus the “true” similarity with these individuals was skewed based on the source of the SNPs chosen for the array. Due to the ability to detect unique novel differences, particularly between the wild accessions, the SSR data set was less skewed and behaved more “realistically” or closer to “true”. While the SSRs and SNP array produce different similarity values, the technologies both provide methods for determining relationships between individuals.

### Genome-wide association analysis for three cottonseed nutritional traits

**Fig. 8:**
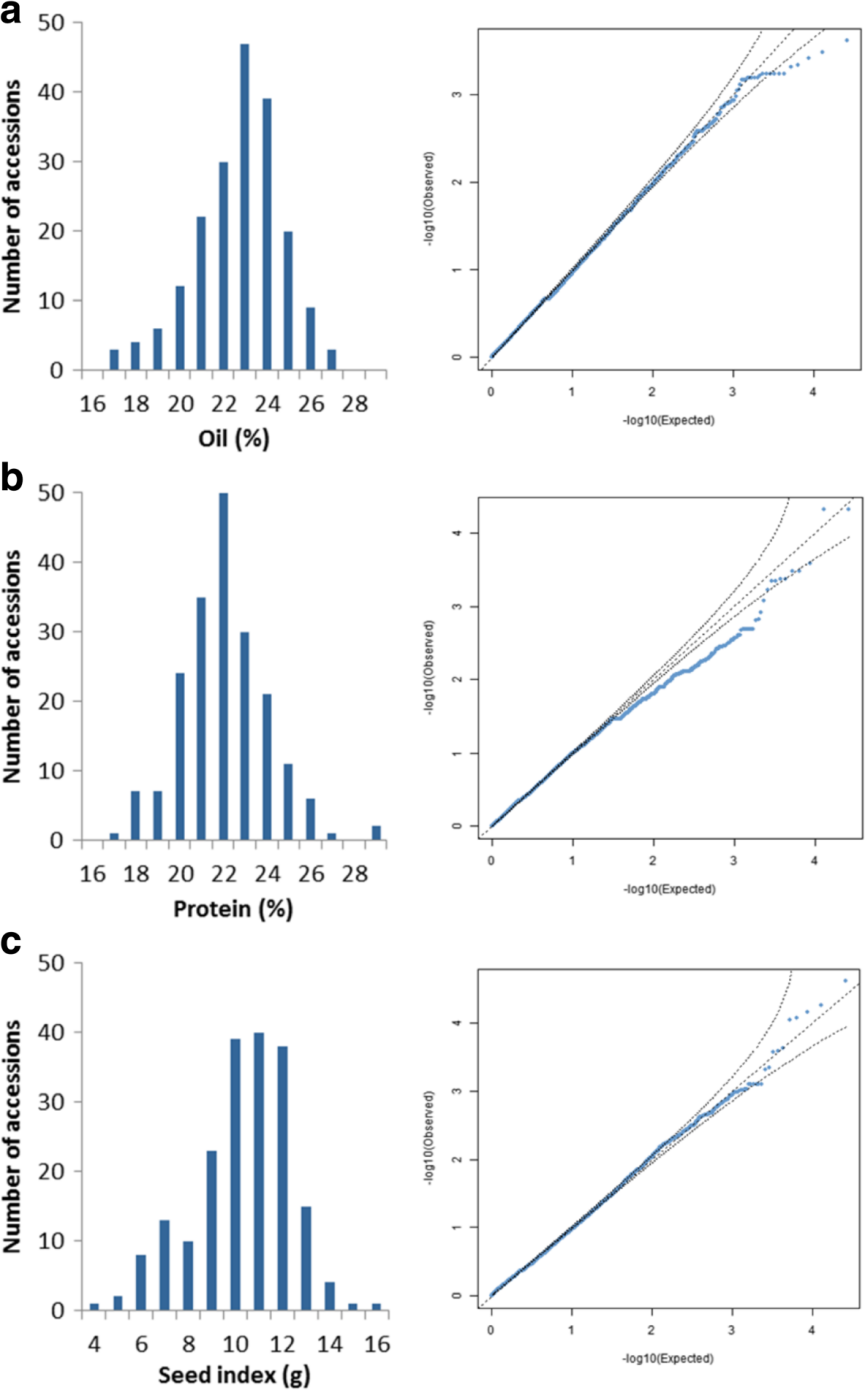
Histogram distributions and Q-Q plots of corrected *p*-values for seed trait association analysis. The corrected *p*-values are based on relatedness for population structure versus the expected *p*-values. GEMMA software adjusted for population structure over 26,099 SNPs for (**a**) seed oil content (%), (**b**) seed protein content (%), and (**c**) seed index (grams per 100 seeds)

Three traits were analyzed for the set of 195 accessions common with Hinze et al. [10] using the Genome-wide Efficient Mixed Model Association (GEMMA) software for detecting QTL. The seed index or weight in grams per 100 seeds was found to have the highest phenotypic variance explained (PVE) with 0.954 (i.e., 95.4% explained), while seed oil content had a moderate PVE of 0.628 and protein content had the lowest PVE of 0.230. Moreover, the estimated standard error for seed index PVE was very low (0.035), whereas the standard errors for both protein and oil content PVE were considerably higher -- 0.117 and 0.149, respectively. All three statistical tests available for analyses in GEMMA were utilized, including the Wald test, likelihood ratio test (LRT) and the score test for each analysis. Corrected *p*-values were analyzed versus expected ratios for each phenotype, and it was observed that the relatedness calculated using genetic similarities was efficient in correcting for population structure (Fig. 8a, b and c). Corrected *p*-values for the three statistics were analyzed using a false discovery rate of 0.05. Whereas analysis of seed oil content and seed index did not produce any significant loci among the 26,099 SNPs, the analysis of seed protein content identified four significant loci under the Wald test and FDR of 0.05 with *p*-value ≤ 3.7e-06 (Table 5): i28873Gh, i34975Gh, i20295Gh, and i22490Gh. Two of the loci, i28873Gh and i34975Gh, remained statistically significant based on a more stringent correction of multiple testing with Bonferroni’s adjustment and were also significant with the LRT and score test statistics with a more relaxed FDR of 0.15 (*p*-value ≤ 1.6e-05).

**Table 5:** Four SNP loci significantly related to protein content as determined by genome-wide association analysis

SNP		Significance test	*p*-value	Genome sequence	Chromosome	Position
Marker	i28873Gh	Wald test	3.70E-06	*G. raimondii* v221	*	0
Group	TAMU	Likelihood Ratio Test	1.11E-05	*G. arboreum* v2	Ca13	41,692,946
Group_name	GH_TBb049I03r236	Score Test	4.81E-05	*G. hirsutum* NBI v1.1	NBI_A02	7,820,104
Sequence	TCGATATGAACGGAAAATGCTTGCTCGTCGGTTGGAAGGGGACGCCGATGYTTTCAATTTCGGTTTGGAA ATTTCTATAAGCCAATGTCTAAATGTTACCA					
Marker	i34975Gh	Wald test	3.70E-06	*G. raimondii* v221	Chr05	7,028,098
Group	TAMU	Likelihood Ratio Test	1.11E-05	*G. arboreum* v2	Ca13	41,671,786
Group_name	GH_TBb119J20f639	Score Test	4.81E-05	*G. hirsutum* NBI v1.1	NBI_A02	7,800,710
Sequence	ATGGATGACAGAAATAGGACTATGATCAATCCCATCCACCGCTACTCGGTMCCTGTGTATCCAGGTACC CAACACAAAGCTAGCTAGCTATGACAGTAAAT					
Marker	i20295Gh	Wald test	1.52E-05	*G. raimondii* v221	Chr13	30,556,525
Group	CSIRO	Likelihood Ratio Test	9.78E-04	*G. arboreum* v2	*	0
Group_name	Scaffold_13_30556525	Score Test	6.41E-03	*G. hirsutum* NBI v1.1	*	0
Sequence	AACGTACTAAATTCGTAGTTAGATAGTAGCCAAGGACTCACTTAAACCAACTAAAACATCAACCTATTCTAAGTTCTCATGTAACAAAAATTTAACATAAYAAACTTAGAATGCTTATAACTCGGTCTATGCTTAACCTTTTCACCTAAAACGAATTTTGTTCACCTATTTAGTCTTCTACGACTAATCATCAACCCTTAA					
Marker	i22490Gh	Wald test	1.60E-05	*G. raimondii* v221	*	0
Group	USDA	Likelihood Ratio Test	1.03E-03	*G. arboreum* v2	*	0
Group_name	CFB2569	Score Test	6.41E-03	*G. hirsutum* NBI v1.1	NBI_D01	35,251,544
Sequence	TGCCGCATACTTGTGGACCACATARTCGTGTACAATTGGAAAATTAGGGATTTAGAGGAATTTTGGTGCC ACACGACCGTGTGGCTGATT					

Information is provided on *p*-values associated with three significance tests, as well as known chromosomal locations in three available *Gossypium* genome sequences

These two most highly significant loci were both derived from BAC-associated SNPs [38]. The marker i28873Gh was originally named GH_TBb059I03r236, and i34975Gh was named GH_TBb119J20f639. Marker i28873Gh was mapped to linkage group “Chr 02” (Chromosome 02) in the high-density intraspecific genetic map [38], and the sequences of both markers aligned to a 7.80–7.82 Mb region of the “A02” scaffold (Chromosome 02) on the NBI *G. hirsutum* draft genome sequence [50]. There are two candidate genes located near these two markers on the genome sequence: 1) Gh_A02G0521, which is an mRNA for the protein phosphatase 4 regulatory subunit 3 which is a suppressor of MEK (SMEK PPP4R3) and 2) Gh_A02G0522, an uncharacterized mRNA. The other two markers that were identified are i20295Gh which is a gene-associated SNP identified on *G. raimondii* scaffold_13_30556525 which does not align to any other *Gossypium* draft sequence [35], and i22490Gh which was identified in a genotyping-by-sequencing analysis by USDA (D.D. Fang) as CFB2569 and aligns to 35.25 Mb region on *G. hirsutum* D01 (Chromosome 15) [54]. The closest gene nearby this region on D01 is 14Kb away and named Gh_D01G1288. This gene is an mRNA for an unnamed coil family protein that has Myb DNA binding characteristics with a Myb_SANT-line domain, related to *Arabidopsis thaliana* protein AT2G24960.2.

## Discussion

The development of next generation sequencing technologies and the resulting detection of thousands of markers, primarily SNPs, have increased the power available to analyze diversity in germplasm collections [21, 55, 56], and to use this diversity to make advances in plant breeding programs [57–61]. We have surveyed variation across *Gossypium* species utilizing the recently developed CottonSNP63K array and a large panel of 390 *Gossypium* samples comprising 292 improved cultivars of *G. hirsutum* and small sets of non-improved *G. hirsutum* and other *Gossypium* species. This data allowed us to appraise the utility of the array for evaluating genetic diversity and population structure, and to conduct SNP-based characterization and analyses on a significant portion of the USDA National Cotton Germplasm Collection. The observed patterns of diversity among *Gossypium* species and within *G. hirsutum* agreed with expectations based on previous studies with molecular markers [4, 10]. The markers distinctly separated *G. hirsutum* from other species and distinguished the wild from improved types of *G. hirsutum*. Within improved types of *G. hirsutum*, the observed clustering may be a sign of slight ascertainment bias as has been noted in other SNP studies [62, 63] with samples similar to TM-1 being localized towards the bottom of the two clusters of improved *G. hirsutum*. We found the array to be effective for grouping germplasm using PCoA and STRUCTURE analyses.

We identified a subset of 192 improved and wild cotton accessions that were run in a previous SSR analysis and on the CottonSNP63K array, and a comparison of grouping and discrimination revealed generally similar patterns for the SSR- versus SNP-based distributions. Moreover, combining the SSR data with the SNP dataset resulted in plots without marked changes, indicating that either technology is sufficient for determining relationships between samples. It is nonetheless noteworthy that the CottonSNP63K array and cluster file were primarily designed to discriminate among cultivated types of *G. hirsutum* and between cultivated *G. hirsutum* types versus non-cultivated germplasm, including several diploid and tetraploid species [38]. The ability to discriminate among non-cultivated types was not an emphasized criterion in selecting which SNPs to place in the array, but the array seems to function reasonably well for that purpose, too. The findings here indicate those objectives were achieved, in that the array and associated cluster file efficiently discerns differences among *G. hirsutum* cultivars, and between improved genotypes from wild forms of *G. hirsutum* and other species. Thus, the SNP array presents a facile means of identifying several thousand or more SNPs between most pairwise combinations of *G. hirsutum* genotypes. Given that the intraspecific linkage maps average about 3,500 cM [38], the expected density of markers for a given pair of parents would typically exceed one SNP per cM.

SNPs have quickly become the preferred marker system for measuring genetic diversity in plants due to the potential to identify a substantial number of SNPs throughout the plant genome and therefore have greater power when associating that information with phenotypic traits of interest. Only recently have SNPs become utilized for genotyping by the cotton research community. To be successful, SNPs need to be dense and polymorphic within the population of interest. The SNPs on the array displayed polymorphism across the range of samples tested. Over 60% of the SNPs in this dataset were found to be polymorphic in the economically important *G. hirsutum* species. This suggests that this array will likely have high utility for discrimination and association studies within the primarily cultivated cotton species. Specifically, discrimination by the array is best for US and Australian cultivars as they are the source of the SNPs used in development of the array. In the present study, we have shown that the SNP genotype information obtained using CottonSNP63K array has enabled GWAS to identify loci associated with a seed trait. In our analysis of cottonseed nutritional traits, four SNPs were found significantly associated with protein content, with two located in Chromosome 2, a member of the A_t_ subgenome. In a recent association mapping study of seed oil and protein in cotton, 228 SSR markers were used to genotype 180 elite *G. hirsutum* cultivars [64]. The SSR-based GWAS detected 12 markers across 9 chromosomes associated with seed protein. Interestingly, chromosome 2 that was identified with multiple individual markers in our SNP-based study was not significant in the SSR study. This may be due to much fewer SSRs tested and the corresponding low marker density relative to the number of effective SNP loci, particularly on chromosome 2. Of the eight SSR loci examined on chromosome 2 by Liu et al. [64], none co-localized with our SNPs based on alignment positions to currently available cotton genome sequences.

While multiple markers with significant associations were identified with seed protein content, no significant associations were found for seed oil content or seed index. Detection of significant marker associations is dependent upon statistical power to detect linkage disequilibrium of the marker with the causative allele for the phenotype. Statistical power in GWAS can be influenced by many factors including trait architecture and effect size, number of samples in the population, number of markers, distribution of markers, statistical method utilized, etc. [65, 66]. It may be that the effect sizes of these traits are extremely small implying a multi-genic effect which combined with a relatively small number of samples for a standard GWAS analysis would cause the inability to detect significant associations with the seed index and oil content phenotypes. The differences in trait architecture of the phenotypes is observed in the coefficient of variations (CV) and showed that the trait architecture of seed index with CV of 20.9% was very different from that of oil content (8.8%) and protein content (9.1%). This large amount of phenotypic variability in the seed index trait of the sampled population could have possibly caused QTL determination of the loci responsible for the trait to be difficult. The variability in the current study likely resulted because the data were not from a single experiment in which all of the 195 accessions were included. Rather, the data were obtained from seed harvested from a number of different seed increases grown in different locations and years [11], as in common in large germplasm collections. A quantitative trait defined in a range of environments is likely to produce different accession values that are confounded by these non-genetic effects, and it is these accession means that were used in the GWAS experiment. Inheritance studies have shown that protein and oil are both influenced by the environment, with protein being more susceptible to these changes [64, 67, 68]. Detection of significant associations with GWAS studies has generally been difficult unless a large number of samples are utilized for lowly heritable traits, as those traits that are determined by a large number of very small effect loci are very difficult to identify. Nonetheless, the utilization of standardized genotyping methods, such as possible with the array, allow for easily adding samples to the population and reanalyzing with the additional data to assist in generating additional power for detection in the future.

In addition to the GWAS performed here, an additional analysis using the CottonSNP63K array, followed by a “targeted” approach of a specific set of SNPs rather than genome-wide SNPs was recently published. Zhu et al. [69] identified a gene on chromosome 15 of the D_t_ subgenome that affects leaf shape in cotton; the findings were supported by similarity to genes regulating leaf morphology in other plant species. A separate study targeting SNPs for genetic male sterility (*ms*) in cotton [70] successfully linked a SNP to the recessive gene *ms5* on chromosome 12 (A12) and *ms6* on chromosome 26 (D12). Similarly, Ellis et al. [71] used the array to identify markers linked to a viral resistance gene for cotton bunchy top disease. This targeted SNP association analysis identified nearly as good an interval spanning the *Cbd* resistance locus as was obtained from screening an F_2:3_ population. These results demonstrate that genotyping and GWAS can be effectively conducted with SNPs in cotton utilizing the array genotyping platform. As with many quantitative traits, statistical power necessary for identification of all loci responsible for a trait needs to be high to detect the small effect of numerous loci. In instances where a large proportion of the overall phenotype is explained by large numbers of loci, genomic selection utilizing the complete genotypes obtained by the array may provide a promising future avenue of research. An additional option would be to increase power by adding additional samples, as has been done with human and other crop analyses. For example, extensive application of an analogous array to the USDA Soybean Germplasm Collection enabled synthesis of haplotype block maps that are expected to lead to the discovery of many SNP-trait associations for economically important characteristics [72]. The consistency and reliability of genotyping with the array is likely to prove valuable in regard to accumulating high-quality genotypic data sets to analyze these kinds of genetic traits, with data potentially being accumulated across multiple labs, large distances and time periods.

We assessed the utility of the CottonSNP63K array data for distinguishing among *G. hirsutum* cultivars by comparing representative cultivars from the NCGC and worldwide cotton researchers. Because breeders often use a limited range of material, assessing the relatedness of cultivars can assist with selecting more distantly related lines to maximize variability in breeding programs. Recent SNP-based reports show a lower diversity in cotton relative to other plant species. A diversity study in soybeans (*Glycine* spp.) showed Chinese landraces of *G. max* L. (H_E_ = 0.338) with a slightly higher diversity than inbreds (H_E_ = 0.313) [73]. Though still showing higher diversity than cotton, an Italian grape (*Vitis* spp.) germplasm collection had higher diversity in the primary cultivated species of *V. vinifera* L. ssp*. sativa* (H_E_ = 0.345) than the wild form, *V. vinifera* L. ssp. *sylvestris* (H_E_ = 0.266) [74]. Our current evaluation with SNPs and previous evaluations with SSRs [10, 25] have revealed differences between individual cultivars with limited differences among groups of cultivars from the United States or from other countries. To maximize genetic diversity estimated with the SNP data presented here, a breeder has multiple options. A breeder could choose parents from those analyzed within this manuscript based on the genome-wide genetic similarity values calculated with all markers (Additional file 5) or could choose two cultivars on opposite ends of either the x- or y-axis shown in the MDS plots (Fig. 4a and b; Additional file 2). If a breeder was interested in genotypes not assayed here but could assign them to a breeding region as defined here, the average expected polymorphisms for a given cross could be estimated based on the category averages. For example, within the US, if germplasm from the eastern region were crossed to germplasm from the plains region, a breeder could expect to observe *ca.* 5,200 polymorphic SNPs between the two samples (Table 2). Breeders interested in utilizing parents developed within the US, could benefit by using cultivars from different regions in a breeding program, as they are generally less similar than cultivars from within the same region. Of particular interest are the US cultivars that could not be assigned to a specific breeding region based on regional location of breeding programs or pedigree (also includes cultivars with mixed origins). Increased heterozygosity/gene diversity and unique alleles were observed among cultivars in this group compared to cultivars with clear origin because this group was comprised of many unique genotypes. The knowledge provided by the screening of germplasm materials will allow for intelligent design of breeding populations to incorporate desired levels of known diversity particularly for targeting or pyramiding regions of interest that may provide multiple modes of action for desirable traits in the future. With continuing advances in SNP analysis technologies, the diversity data regarding specific pairs or groups of parents could also be used in concert with genetic maps to identify and select ad hoc subsets of markers to deploy for analysis and/or selection of specific families or populations. Many breeders thus might consider running the CottonSNP63K array against their breeding lines to assess their germplasm relative to other germplasm of interest, and select marker subset(s) that will prospectively maintain effectiveness but reduce genotyping costs.

While the current study is one of the first looking primarily at US germplasm, SNPs have been tested to determine their suitability in the examination of distinctness, uniformity and stability (DUS) for cotton varieties in China [75]. DUS examination typically involves a grow-out test with measurement of a set of phenotypic observations; however, the more rapid results provided by molecular markers have been allowed under the International Union for the Protection of New Varieties of Plants (UPOV) system of plant variety protection in specific situations [76]. Twenty-three core SNP markers were identified by Kuang et al. [75] that clustered 30 standard cotton cultivars into groups consistent with known pedigrees. A similar effort was undertaken to verify marker-based distinctness for registering alfalfa cultivars [77]. They compared 2,902 SNP markers with 11 morpho-physiological measures and 41 polymorphic SSRs on a set of 11 alfalfa landrace cultivars. Even though the authors identified inconsistencies for SSR and SNP markers with morpho-physiological traits, they assert that marker-based assays have high potential to substitute for, or complement, morphological distinctness measures in alfalfa cultivars. Many of these are the same issues that arise when officially registering cultivars of any crop and are applicable to identifying accessions in germplasm collections.

The integration of genomic data into germplasm collection documentation systems and its combination with taxonomic and phenotypic data will impact how these genetic resources are conserved as well as how they are used by plant breeders. In the current study, eight pairs of genotypes with high IBS values (putative duplicates based on genotype) were grown in the field and phenotypic descriptors were measured. Results of genotype and phenotype comparisons were mixed. In some cases, cultivars with a highly similar DNA profile for this set of SNP markers also had identical phenotypes, while in other cases, cultivars with a highly similar DNA profile had multiple phenotypic differences. The observed phenotypic differences are too strong to be due to natural environmental effects. It must be noted that unusual observations may be due to the possibility of human errors in any assessments, whether they be based on genotype or phenotype. Therefore, a researcher desiring to use a similar approach to remove highly similar individuals from their lines may wish to verify a small number of markers on independent DNA isolations with simplex SNP marker assays in the lab to ensure that a mistake was not made in sampling prior to more extensive field-based assessment. Based on our data at this time, we assert that any potential duplicates or redundancies within a collection cannot be ascertained solely by genotype or phenotype profiles, but that SNP and/or SSR data can bring greater power for identification of redundancy, solely due to the number of “markers or traits” one can look at. Often with phenotypic characterization, one is only working with 50–75 traits at most. SNP markers may be more appropriate to distinguish among closely related individuals while SSR markers can easily estimate diversity among less related germplasm. When comparing SSR and SNP markers in cotton cultivars, the study of Kuang et al. [75] found that SSR markers tend to correlate cultivars with their geographic origin while SNP markers more consistently correlate cultivars based on kinship or pedigree; however, no rationale was provided to explain this difference. Transcriptome profiles of lines could also potentially be different without causing an easily detectable phenotype in the field and the causal allele in most cases may not be fully represented on the marker profiles generated with the array, so a researcher may also wish to study the transcriptomic profiles of lines in the future prior to concluding lines are redundant. Until more thorough studies are available, a complement of genetic and phenotypic data should be analyzed prior to declaring two accessions as genetically distinct or duplicates.

## Conclusions

With increasing SNP discovery projects and the development of the CottonSNP63K array to assay thousands of SNPs, it is anticipated that SNP markers will play an increasingly important role in cotton genetics, germplasm conservation, and breeding applications. In this study, we provide a large genome-wide variation data set for primarily cultivated cotton. Thousands of SNPs in representative cotton genotypes provide an opportunity to finely discriminate among cultivated cotton from around the world. The SNPs will be useful as dense markers of genome variation for association mapping approaches aimed at correlating molecular polymorphisms with variation in phenotypic traits, as well as for molecular breeding approaches in cotton.

## Additional files

Additional file 1: Results and discussion in relation to the removal of admixed/misclassified samples. Five samples were removed from overall SNP diversity analysis, and three samples were removed from the comparison of SNP and SSR data. This file presents the original results and discusses why the samples were subsequently removed. For the original diversity analysis, this file includes the MDS figures, Venn diagrams of unique and shared SNPs, and distribution of pairwise IBS values when these samples are included. Likewise, for the comparison of SNP and SSR data, the original principal coordinate analyses and the plots comparing SNP- and SSR-based genetic similarity are shown. (DOCX 325 kb)
Additional file 2: List of *Gossypium* samples genotyped for SNP diversity analyses. The table includes information for seed source, improved/wild classification, assigned categories for improved breeding regions, assigned categories for wild race types, availability of SSR genotypes for SNP-SSR comparison, and values for seed oil, protein, and seed index for samples used in GWAS analysis. In addition, for each sample, the coordinates of the first two MDS dimensions are listed for Figs. 3 and 4a, b. Five samples are included here that were identified as outliers and not used in the SNP analysis. Siokra 104–90 was not a cultivar developed in Australia (I. Wilson, personal communication). It may be a mislabeling of Siokra 1–4/649 which is actually just Siokra 1–4. (XLSX 97 kb)
Additional file 3: Individual matrices with numbers of different and identical SNPs for entries with the same name. These values were extracted directly from Additional file 5. Values above the diagonal represent the count of homozygous differences between pairs of samples. Values below the diagonal represent the number of identical SNPs genotyped between pairs. Heterozygous SNPs within samples were not counted in the number of differences between samples. SNP numbers were determined using all SNPs on the CottonSNP63K array. Five samples are included here that were identified as outliers and not used in the diversity analysis. (XLSX 17 kb)
Additional file 4: Distribution of SNP minor allele frequency across groups of germplasm. Categories of minor allele frequency are used to show the relative polymorphism of SNPs within a set of germplasm. The values shown are the fraction of all SNPs within *Gossypium* spp., *G. hirsutum*, cultivated and wild types of *G. hirsutum*, US and non-US cultivated types, and cultivated types within US breeding regions. (XLSX 16 kb)
Additional file 5: Matrix showing numbers of different and identical SNPs for all pairwise combinations of 395 samples. Values above the diagonal represent the count of homozygous differences between pairs of samples. Values below the diagonal represent the number of identical SNPs genotyped between pairs. Heterozygous SNPs within samples were not counted in the number of differences between samples. SNP numbers were determined using all SNPs on the CottonSNP63K array. Five samples are included here that were identified as outliers and not used in the diversity analysis. (XLSX 1032 kb)

## Abbreviations

FDR: False discovery rate
GDRS: *Gossypium* Diversity Reference Set
GEMMA: Genome-wide Efficient Mixed Model Association
IBS: Identical by state
LRT: Likelihood ratio test
MAF: Minor allele frequency
MDS: Multidimensional scaling
NCGC: National Cotton Germplasm Collection
PCoA: Principal coordinate analysis

## References

[1] Alford BB, Liepa GU, Vanbeber AD (1996). Cottonseed protein: What does the future hold?. Plant Foods Hum Nutr.

[2] Rathore KS, Sunilkumar G, Campbell LAM (2011). Cotton plant with seed-specific reduction in gossypol.

[3] Campbell B, Saha S, Percy R, Frelichowski J, Jenkins J, Park W, Mayee C, Gotmare V, Dessauw D, Giband M, Du X, Jia Y, Constable G, Dillon S, Abdurakhmonov I, Abdukarimov A, Rizaeva S, Adullaev A, Barroso P, Padua J, Hoffmann L, Podolnaya L (2010). Status of the global cotton germplasm resources. Crop Sci.

[4] Hinze L, Fang D, Gore M, Scheffler B, Yu J, Frelichowski J, Percy R (2015). Molecular characterization of the *Gossypium* Diversity Reference Set of the US National Cotton Germplasm Collection. Theor Appl Genet.

[5] Hutchinson JB (1951). Intra-specific differentiation in *Gossypium hirsutum*. Heredity.

[6] Brubaker CL, Wendel JF (1994). Reevaluating the origin of domesticated cotton (*Gossypium hirsutum*; Malvaceae) using nuclear restriction fragment length polymorphisms (RFLPs). Am J Bot.

[7] D’Eeckenbrugge G, Lacape J (2014). Distribution and differentiation of wild, feral, and cultivated populations of perennial Upland cotton (*Gossypium hirsutum* L.) in Mesoamerica and the Caribbean. PLoS One.

[8] Grover C, Zhu X, Grupp K, Jareczek J, Gallagher J, Szadkowski E, Seijo J, Wendel J (2015). Molecular confirmation of species status for the allopolyploid cotton species, *Gossypium ekmanianum* Wittmack. Genet Res Crop Evol.

[9] Lubbers EL, Chee PW, Paterson AH (2009). The worldwide gene pool of *G. hirsutum* and its improvement. Genetics and genomics of cotton.

[10] Hinze LL, Gazave E, Gore MA, Fang DD, Scheffler BE, Yu JZ, Jones DC, Frelichowski J, Percy RG (2016). Genetic diversity of the two commercial tetraploid cotton species in the *Gossypium* Diversity Reference Set. J Hered.

[11] Hinze LL, Horn PJ, Kothari N, Dever JK, Frelichowski J, Chapman KD, Percy RG (2015). Nondestructive measurements of cottonseed nutritional trait diversity in the US National Cotton Germplasm Collection. Crop Sci.

[12] Abdurakhmonov I, Kohel R, Yu J, Pepper A, Abdullaev A, Kushanov F, Salakhutdinov I, Buriev Z, Saha S, Scheffler B, Jenkins J, Abdukarimov A (2008). Molecular diversity and association mapping of fiber quality traits in exotic *G. hirsutum* L. germplasm. Genomics.

[13] Abdurakhmonov I, Saha S, Jenkins J, Buriev Z, Shermatov S, Scheffler B, Pepper A, Yu J, Kohel R, Abdukarimov A (2009). Linkage disequilibrium based association mapping of fiber quality traits in G. hirsutum L. variety germplasm. Genetica.

[14] Lacape J, Dessauw D, Rajab M, Noyer J, Hau B (2007). Microsatellite diversity in tetraploid *Gossypium* germplasm: assembling a highly informative genotyping set of cotton SSRs. Mol Breed.

[15] Fang D, Hinze L, Percy R, Li P, Deng D, Thyssen G (2013). A microsatellite-based genome-wide analysis of genetic diversity and linkage disequilibrium in Upland cotton (*Gossypium hirsutum* L.) cultivars from major cotton-growing countries. Euphytica.

[16] Zhao Y, Wang H, Chen W, Li Y, Gong H, Sang X, Huo F, Zeng F (2015). Genetic diversity and population structure of elite cotton (*Gossypium hirsutum* L.) germplasm revealed by SSR markers. Plant Syst Evol.

[17] Tyagi P, Gore M, Bowman D, Campbell B, Udall J, Kuraparthy V (2014). Genetic diversity and population structure in the US Upland cotton (*Gossypium hirsutum* L.). Theor Appl Genet.

[18] Tu J, Zhang M, Wang X, Zhang X, Lin Z (2014). Genetic dissection of upland cotton (*Gossypium hirsutum*) cultivars developed in Hubei Province by mapped SSRs. Genet Mol Res.

[19] Elci E, Akiscan Y, Akgol B (2014). Genetic diversity of Turkish commercial cotton varieties revealed by molecular markers and fiber quality traits. Turkish J Bot.

[20] Jena SN, Srivastava A, Singh UM, Roy S, Banerjee N, Mohan Rai K, Singh SK, Kumar V, Chaudhary LB, Roy JK, Tuli R, Sawant SV (2011). Analysis of genetic diversity, population structure and linkage disequilibrium in elite cotton (*Gossypium* L.) germplasm in India. Crop Pasture Sci.

[21] Kilian B, Graner A (2012). NGS technologies for analyzing germplasm diversity in genebanks. Brief Funct Genomics.

[22] McCouch SR, McNally KL, Wang W, Sackville Hamilton R (2012). Genomics of gene banks: A case study in rice. Am J Bot.

[23] Korir NK, Han J, Shangguan L, Wang C, Kayesh E, Zhang Y, Fang J (2013). Plant variety and cultivar identification: Advances and prospects. Crit Rev Biotechnol.

[24] McGregor SE (1976). Crop plants and exotic plants. In: Insect pollination of cultivated crop plants. Agricultural Research Service, US Department of Agriculture.

[25] Hinze L, Dever J, Percy R (2012). Molecular variation among and within improved cultivars in the U.S. Cotton Germplasm Collection. Crop Sci.

[26] Hamblin MT, Warburton ML, Buckler ES (2007). Empirical comparison of simple sequence repeats and single nucleotide polymorphisms in assessment of maize diversity and relatedness. PLoS One.

[27] Goddard M (2009). Genomic selection: Prediction of accuracy and maximisation of long term response. Genetica.

[28] Kaur S, Francki MG, Forster JW (2012). Identification, characterization and interpretation of single-nucleotide sequence variation in allopolyploid crop species. Plant Biotechnol J.

[29] Byers RL, Harker DB, Yourstone SM, Maughan PJ, Udall JA (2012). Development and mapping of SNP assays in allotetraploid cotton. Theor Appl Genet.

[30] Ashrafi H, Hulse-Kemp AM, Wang F, Yang SS, Guan X, Jones DC, Matvienko M, Mockaitis K, Chen ZJ, Stelly DM, Van Deynze A (2015). A long-read transcriptome assembly of cotton (*Gossypium hirsutum* L.) and intraspecific single nucleotide polymorphism discovery. Plant Genome.

[31] Hulse-Kemp AM, Ashrafi H, Zheng XT, Wang F, Hoegenauer KA, Maeda ABV, Yang SS, Stoffel K, Matvienko M, Clemons K, Udall JA, Van Deynze A, Jones DC, Stelly DM (2014). Development and bin mapping of gene-associated interspecific SNPs for cotton (*Gossypium hirsutum* L.) introgression breeding efforts. BMC Genomics.

[32] Chen W, Yao JB, Chu L, Li Y, Guo XM, Zhang YS (2014). The development of specific SNP markers for chromosome 14 in cotton using next-generation sequencing. Plant Breed.

[33] Li XM, Gao WH, Guo HL, Zhang XL, Fang DD, Lin ZX (2014). Development of EST-based SNP and InDel markers and their utilization in tetraploid cotton genetic mapping. BMC Genomics.

[34] Rai KM, Singh SK, Bhardwaj A, Kumar V, Lakhwani D, Srivastava A, Jena SN, Yadav HK, Bag SK, Sawant SV (2013). Large-scale resource development in *Gossypium hirsutum* L. by 454 sequencing of genic-enriched libraries from six diverse genotypes. Plant Biotechnol J.

[35] Zhu Q-H, Spriggs A, Taylor JM, Llewellyn D, Wilson I (2014). Transcriptome and complexity-reduced, DNA-based identification of intraspecies single-nucleotide polymorphisms in the polyploid *Gossypium hirsutum* L. G3. Genes Genom Genet.

[36] Logan-Young CJ, Yu JZ, Verma SK, Percy RG, Pepper AE (2015). SNP discovery in complex allotetraploid genomes (*Gossypium* spp., *Malvaceae*) using genotyping by sequencing. Appl. Plant Sci.

[37] Van Deynze A, Stoffel K, Lee M, Wilkins T, Kozik A, Cantrell R, Yu J, Kohel R, Stelly D (2009). Sampling nucleotide diversity in cotton. BMC Plant Biol.

[38] Hulse-Kemp AM, Lemm J, Plieske J, Ashrafi H, Buyyarapu R, Fang DD, Frelichowski J, Giband M, Hague S, Hinze LL, Kochan KJ, Riggs PK, Scheffler JA, Udall JA, Ulloa M, Wang SS, Zhu Q-H, Bag SK, Bhardwaj A, Burke JJ, Byers RL, Claverie M, Gore MA, Harker DB, Islam MS, Jenkins JN, Jones DC, Lacape J-M, Llewellyn DJ, Percy RG (2015). Development of a 63K SNP array for cotton and high-density mapping of intraspecific and interspecific populations of *Gossypium* spp. G3. Genes Genom Genet.

[39] de Menezes I, Barroso P, Hoffmann L, Lucena V, Giband M (2010). Genetic diversity of mocó cotton (*Gossypium hirsutum* race *marie-galante*) from the northeast of Brazil: Implications for conservation. Botany-Botanique.

[40] Chang C, Chow C, Tellier L, Vattikuti S, Purcell S, Lee J (2015). Second-generation PLINK: Rising to the challenge of larger and richer datasets. GigaScience.

[41] Purcell S, Neale B, Todd-Brown K, Thomas L, Ferreira MAR, Bender D, Maller J, Sklar P, de Bakker PIW, Daly MJ, Sham PC (2007). PLINK: A tool set for whole-genome association and population-based linkage analyses. Am J Hum Genet.

[42] Danecek P, Auton A, Abecasis G, Albers CA, Banks E, DePristo MA, Handsaker RE, Lunter G, Marth GT, Sherry ST, McVean G, Durbin R, Group GPA (2011). The variant call format and VCFtools. Bioinformatics.

[43] Raj A, Stephens M, Pritchard JK (2014). fastSTRUCTURE: Variational inference of population structure in large SNP data sets. Genetics.

[44] Hinze LL, Gazave E, Gore MA, Fang DD, Scheffler BE, Yu JZ, Jones DC, Frelichowski J, Percy RG. Data from: Genetic diversity of the two commercial tetraploid cotton species in the Gossypium Diversity Reference Set. In.: Dryad Data Repository. http://dx.doi.org/10.5061/dryad.0bn55; 2016. 10.1093/jhered/esw004 PMC4885238 26774060

[45] Jaccard P (1908). Nouvelles recherches sur la distribution florale. Bull Soc Vaud Sci Nat.

[46] Rohlf FJ (2000). NTSYSpc: Numerical Taxonomy and Multivariate Analysis System, version 2.2.

[47] Zhou X, Stephens M (2012). Genome-wide efficient mixed-model analysis for association studies. Nat Genet.

[48] Benjamini Y, Hochberg Y (1995). Controlling the false discovery rate: A practical and powerful approach to multiple testing. J R Stat Soc Series B (Methodological).

[49] Paterson AH, Wendel JF, Gundlach H, Guo H, Jenkins J, Jin DC, Llewellyn D, Showmaker KC, Shu SQ, Udall J, Yoo MJ, Byers R, Chen W, Doron-Faigenboim A, Duke MV, Gong L, Grimwood J, Grover C, Grupp K, Hu GJ, Lee TH, Li JP, Lin LF, Liu T, Marler BS, Page JT, Roberts AW, Romanel E, Sanders WS, Szadkowski E (2012). Repeated polyploidization of *Gossypium* genomes and the evolution of spinnable cotton fibres. Nature.

[50] Zhang T, Hu Y, Jiang W, Fang L, Guan X, Chen J, Zhang J, Saski CA, Scheffler BE, Stelly DM, Hulse-Kemp AM, Wan Q, Liu B, Liu C, Wang S, Pan M, Wang Y, Wang D, Ye W, Chang L, Zhang W, Song Q, Kirkbride RC, Chen X, Dennis E, Llewellyn DJ, Peterson DG, Thaxton P, Jones DC, Wang Q (2015). Sequencing of allotetraploid cotton (*Gossypium hirsutum* L. acc. TM-1) provides a resource for fiber improvement. Nat Biotechnol.

[51] Ladizinsky G (1985). Founder effect in crop-plant evolution. Econ Bot.

[52] Gallagher JP, Grover CE, Rex K, Moran M, Wendel JF. A new species of cotton from the Wake Atoll, Gossypium stephensii (Malvaceae). Syst Bot 2016; (in press).

[53] Kohel RJ, Richmond TR, Lewis CF (1970). Texas Marker-1: Description of a genetic standard for *Gossypium hirsutum* L. Crop Sci.

[54] Islam MS, Thyssen GN, Jenkins JN, Fang DD (2015). Detection, validation, and application of genotyping-by-sequencing based single nucleotide polymorphisms in Upland cotton. Plant Genome.

[55] van Treuren R, van Hintum TJL (2014). Next-generation genebanking: Plant genetic resources management and utilization in the sequencing era. Plant Genet Res.

[56] Mason AS, Zhang J, Tollenaere R, Vasquez Teuber P, Dalton-Morgan J, Hu L, Yan G, Edwards D, Redden R, Batley J (2015). High-throughput genotyping for species identification and diversity assessment in germplasm collections. Mol Ecol Resour.

[57] Rafalski JA (2010). Association genetics in crop improvement. Curr Opin Plant Biol.

[58] Varshney RK, Nayak SN, May GD, Jackson SA (2009). Next-generation sequencing technologies and their implications for crop genetics and breeding. Trends Biotechnol.

[59] Mammadov J, Aggarwal R, Buyyarapu R, Kumpatla S (2012). SNP markers and their impact on plant breeding. Intl J Plant Genomics.

[60] Poland JA, Rife TW (2012). Genotyping-by-sequencing for plant breeding and genetics. Plant Genome.

[61] Thomson MJ (2014). High-throughput SNP, genotyping to accelerate crop improvement. Plant Breed Biotechnol.

[62] Frascaroli E, Schrag T, Melchinger A (2013). Genetic diversity analysis of elite European maize (*Zea mays* L.) inbred lines using AFLP, SSR, and SNP markers reveals ascertainment bias for a subset of SNPs. Theor Appl Genet.

[63] Moragues M, Comadran J, Waugh R, Milne I, Flavell AJ, Russell JR (2010). Effects of ascertainment bias and marker number on estimations of barley diversity from high-throughput SNP genotype data. Theor Appl Genet.

[64] Liu G, Mei H, Wang S, Li X, Zhu X, Zhang T (2015). Association mapping of seed oil and protein contents in upland cotton. Euphytica.

[65] Ball RD, Gondro C, van der Werf J, Hayes B (2013). Genome-Wide Association Studies and Genomic Prediction.

[66] Klein RJ (2007). Power analysis for genome-wide association studies. BMC Genet.

[67] Campbell BT, Chapman KD, Sturtevant D, Kennedy C, Horn P, Chee PW, Lubbers E, Meredith WR, Johnson J, Fraser D, Jones DC (2016). Genetic analysis of cottonseed protein and oil in a diverse cotton germplasm. Crop Sci.

[68] Kothari N, Campbell BT, Dever JK, Hinze LL (2016). Combining ability and performance of cotton germplasm with diverse seed oil content. Crop Sci.

[69] Zhu Q-H, Zhang J, Liu D, Stiller W, Liu D, Zhang Z, Llewellyn D, Wilson I (2016). Integrated mapping and characterization of the gene underlying the okra leaf trait in *Gossypium hirsutum* L. J Exp Bot.

[70] Feng X, Keim D, Wanjugi H, Coulibaly I, Fu Y, Schwarz J, Huesgen S, Cho S (2015). Development of molecular markers for genetic male sterility in *Gossypium hirsutum*. Mol Breed.

[71] Ellis MH, Stiller WN, Phongkham T, Tate WA, Gillespie VJ, Gapare WJ, Zhu Q-H, Llewellyn DJ, Wilson IW (2016). Molecular mapping of bunchy top disease resistance in *Gossypium hirsutum* L. Euphytica.

[72] Song Q, Hyten DL, Jia G, Quigley CV, Fickus EW, Nelson RL, Cregan PB (2015). Fingerprinting soybean germplasm and its utility in genomic research. G3. Genes Genom Genet.

[73] Li Y-H, Li W, Zhang C, Yang L, Chang R-Z, Gaut BS, Qiu L-J (2010). Genetic diversity in domesticated soybean (*Glycine max*) and its wild progenitor (*Glycine soja*) for simple sequence repeat and single-nucleotide polymorphism loci. New Phytol.

[74] Emanuelli F, Lorenzi S, Grzeskowiak L, Catalano V, Stefanini M, Troggio M, Myles S, Martinez-Zapater J, Zyprian E, Moreira F, Grando MS (2013). Genetic diversity and population structure assessed by SSR and SNP markers in a large germplasm collection of grape. BMC Plant Biol.

[75] Kuang M, Ma ZY, Wei SJ, Wang YQ, Zhou DY, Ma L, Fang D, Yang WH (2016). Development of a core set of SNP markers for the identification of upland cotton cultivars in China. J Integr Agric.

[76] UPOV (2013). Guidance on the use of biochemical and molecular markers in the examination of distinctness, uniformity and stability (DUS).

[77] Annicchiarico P, Nazzicari N, Ananta A, Carelli M, Wei Y, Brummer EC (2016). Assessment of cultivar distinctness in alfalfa: a comparison of genotyping-by-sequencing, simple-sequence repeat marker, and morphophysiological observations. Plant Genome.

