# Supplementary material for: Diversity analysis of cotton (Gossypium hirsutum L.) germplasm using the CottonSNP63K Array
Source: BMC Plant Biol. 2017 Feb 3;17:37. doi: 10.1186/s12870-017-0981-y (PMC5291959; doi:10.1186/s12870-017-0981-y)
Supplement: Additional file 1: — Results and discussion in relation to the removal of admixed/misclassified samples. Five samples were removed from overall SNP diversity analysis, and three samples were removed from the comparison of SNP and SSR data. This file presents the original results and discusses why the samples were subsequently removed. For the original diversity analysis, this file includes the MDS figures, Venn diagrams of unique and shared SNPs, and distribution of pairwise IBS values when these samples are included. Likewise, for the comparison of SNP and SSR data, the original principal coordinate analyses and the plots comparing SNP- and SSR-based genetic similarity are shown. (DOCX 325 kb) [file 12870_2017_981_MOESM1_ESM.docx]

**Additional file 1. Results and discussion in relation to the removal of admixed/misclassified samples.** Five samples were removed from overall SNP diversity analysis, and three samples were removed from the comparison of SNP and SSR data. This file presents the original results and discusses why the samples were subsequently removed. For the original diversity analysis, this file includes the MDS figures, Venn diagrams of unique and shared SNPs, and distribution of pairwise IBS values when these samples are included. Likewise, for the comparison of SNP and SSR data, the original principal coordinate analyses and the plots comparing SNP- and SSR-based genetic similarity are shown.

**Genetic similarity and relationships between *Gossypium* breeding groups**

It is noteworthy that five samples representing two genotypes categorized as improved *G. hirsutum* were excluded from analysis because they deviated largely from *G. hirsutum* and particularly cultivated *G. hirsutum* based on their unexpected positions in the MDS plot (Additional file 2; Additional Figure A1). These samples encompassed a NCGC accession designated ‘S-6524’ (SA-2572; PI 630195) and the collaborator-provided sample ‘MCU-5’. Removal of these samples did not appear to affect the MDS plot (Figure 3); however; it had notable influence on the number of unique SNPs in the comparison of improved and wild samples as well as the comparison of improved samples from the US and other countries (Additional Figures A2 and A3; Additional Table A1). These samples likely have admixed genetic backgrounds, and we decided to exclude them from all analyses as they appeared to be significant outliers. Our further phenotyping results indicated that the S-6524 sample appeared to be unusual for a *G. hirsutum* cultivar developed in the United States but similar to germplasm from the N.I. Vavilov Research Institute of Plant Industry (VIR) and Uzbekistan breeding programs (cluster fruiting; hairy stems and leaves) (R. Percy, personal observation). This accession was developed in Uzbekistan and likely had some introgression from *G. barbadense* as suggested by our genomic data. The MCU-5 cultivar (represented here by four technical replicates) was developed in India from a broad pedigree and has been identified as having resistance to Australian races of *Fusarium oxysporum* f. sp. *vasinfectum* [1, 2]. The diverse parentage of this line (“a multi-line cross between Indian Cambodia-type cultivars and cultivars from East Africa, the West Indies and the US, including some contribution from *G. barbadense*” [2]) likely presented a unique combination of alleles that could make this cultivar seem more like a wild type at the genomic level. Putative introgression, admixture, and/or misclassification have been reported previously in cotton diversity studies [3, 4]. Some of these inconsistencies in classification could be due to the extensive morphological diversity observed in *G. hirsutum*. This diversity has led to continued taxonomic reclassifications with the most recent being the resurrection and subsequent molecular confirmation of *G. ekmanianum* as an independent species, rather than a wild type of *G. hirsutum* [5].

**Comparison between SNP and SSR markers in cotton**

Three improved *G. hirsutum* accessions ‘RN96625’ (SA-3493; PI 636104), ‘PD 3’ (SA-3778; PI 511353), and ‘Stoneville 453’ (SA-3749; PI 601544) common to both SNP and SSR datasets were excluded from the final analysis. These improved accessions were noted to be admixed with wild accessions in a principal coordinate analysis based on SSR genotypes (Additional Figure A4b) but not in an analysis based on SNP genotypes (Additional Figure A4a). The SSR-based PCoA comparing only improved accessions showed these accessions behaving more like improved accessions (Additional Figure A4d), and the SNP-based PCoA did not show any anomalies (Additional Figure A4c). Comparison of SSR- and SNP-based genetic similarities showed all pairwise combinations involving these accessions as unique clusters separated vertically from the improved accessions (Additional Figure A5a, b). This is further support for using multiple lines of evidence to define a cultivar based on genotype or phenotype, as two methods may give conflicting results. We should also note that errors may have been introduced with the two biologically different DNA sources used for genotyping SSRs and SNPs.

Additional Table A1. Number of unique SNPs when global breeding regions are evaluated independently, including comparison of the effect of including the five outlier samples from Australia and central Asia.

| Global Breeding Regions | N | *G. hirsutum*, improved N = 292 samples | N | *G. hirsutum*, improved N = 297 samples (including outliers) |
| --- | --- | --- | --- | --- |
| Australia | 18 | 149 | 22 | 277 |
| central Asia | 9 | 76 | 10 | 4702 |
| China/southeast Asia | 8 | 19 | 8 | 4 |
| Europe | 10 | 22 | 10 | 5 |
| Mexico/Central America | 1 | 3 | 1 | 2 |
| n/a | 10 | 376 | 10 | 119 |
| northern Africa | 13 | 118 | 13 | 49 |
| South America | 26 | 36 | 26 | 21 |
| southern Africa | 12 | 54 | 12 | 16 |
| United States | 185 | 1436 | 185 | 461 |

Comparison of unique SNPs found in each of nine global breeding regions plus an “n/a” category for those with either unknown or mixed origins. Most breeding regions are represented by <26 samples while the United States is represented by 185 samples. Therefore, one must be careful to consider the unequal representation when making comparisons among breeding regions.

**Additional Figure A1**. Two dimensional multidimensional scaling (MDS) plot of *Gossypium* samples colored by *G. hirsutum* improved and wild types and non-*G. hirsutum* species. Identical by state genetic similarities of 395 *Gossypium* samples were used in generating the MDS plot. Five outlier samples are circled.


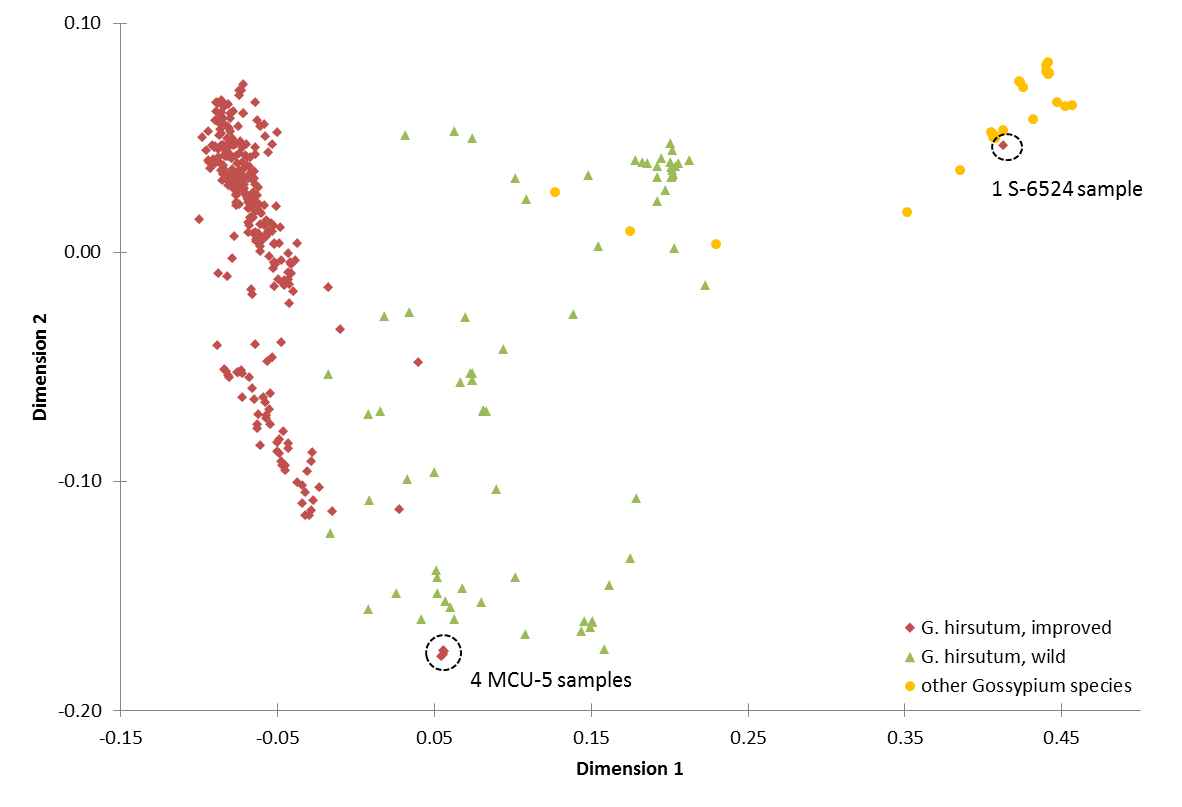


**Additional Figure A2**. Observed changes in comparisons of improved types from the United States with other countries including all five outliers (one S-6524 and four MCU-5 samples) and reanalyzing the SNP data: Venn diagram of unique and shared SNPs a) between improved and wild groups and b) between US and other countries and distributions of pair-wise identical by state (IBS) values for samples from c) improved and wild groups and d) for US and other countries.

a).


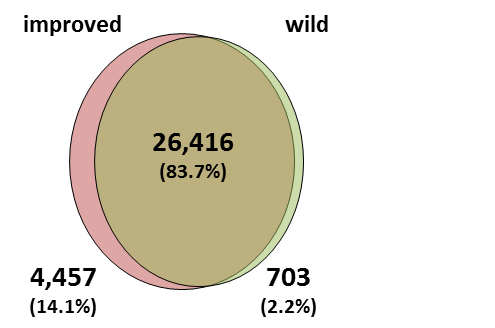


c).


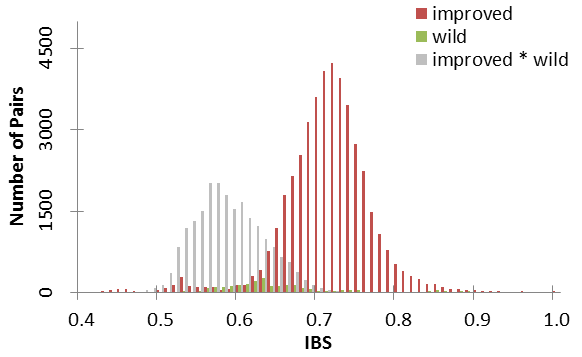


b).


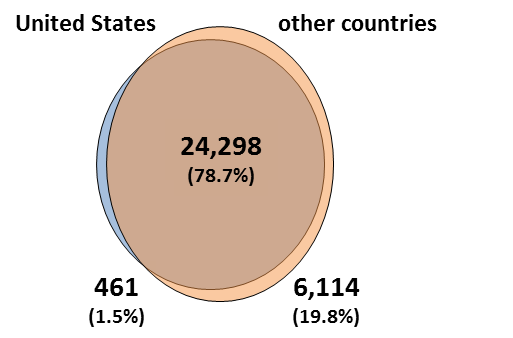


d).


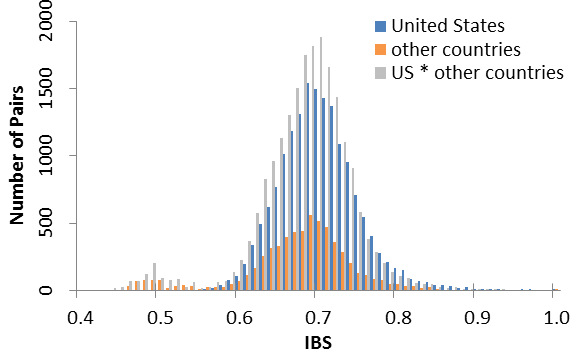


**Additional Figure A3**. Observed changes in comparisons of improved types from the United States with other countries after removing the outlier (a, b) S-6524 sample or (c, d) the four MCU-5 samples and reanalyzing the SNP data: a, c) Venn diagrams of unique and shared SNPs and b, d) distributions of pair-wise identical by state genetic similarities.

a).


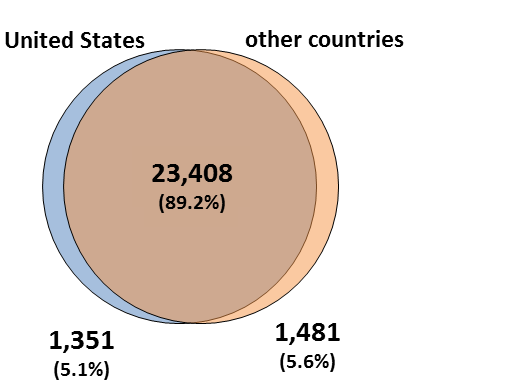


c).


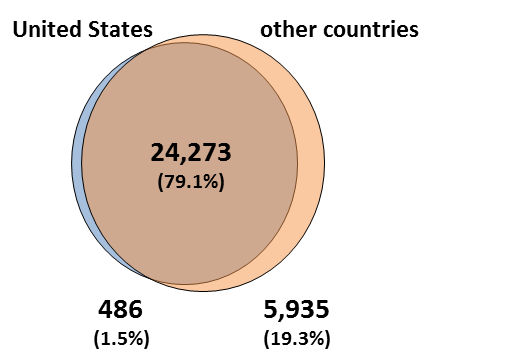
b).


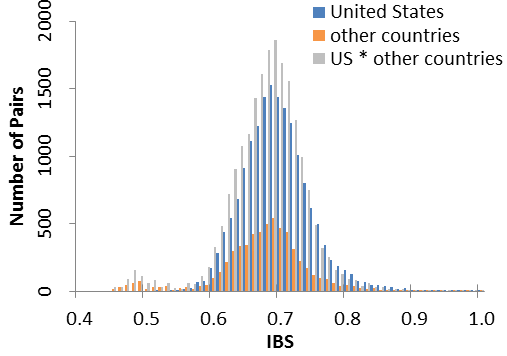


d).


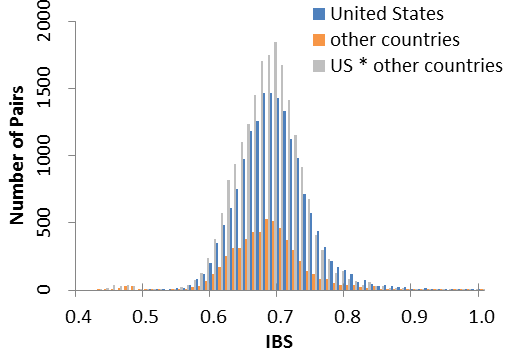


**Additional Figure A4**. Comparison of SNP and SSR principal coordinate analyses based on Jaccard’s coefficient for 195 *G. hirsutum* samples (126 improved and 69 wild types) from the US National Cotton Germplasm Collection based on a) 38,822 SNP loci and b) 105 SSR loci. The 126 improved *G. hirsutum* samples only (80 from the United States and 46 from other countries) were further independently analyzed using c) SNP and d) SSR loci. The three improved *G. hirsutum* samples removed from the final analysis are circled and labeled in each figure.

a).


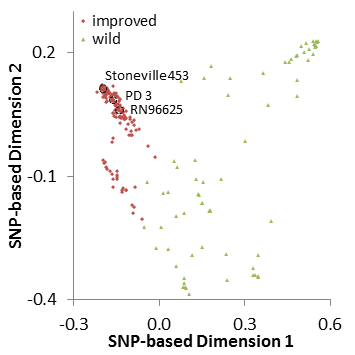


c).


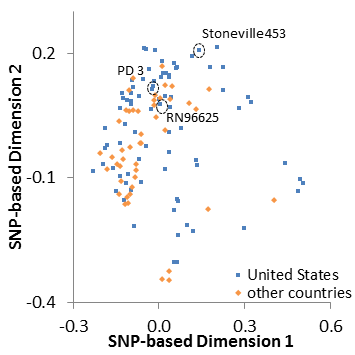


b).


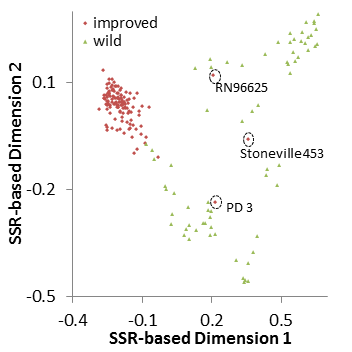


d).


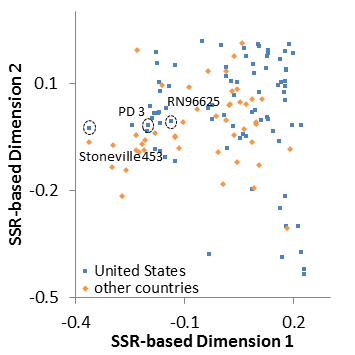


**Additional Figure A5**. Plotting the relationship between SNP (x-axis) and SSR (y-axis) marker sets as calculated using Jaccard’s genetic similarity for a) 195 *G. hirsutum* improved and wild samples (Mantel *r* = 0.763) and b) 126 improved *G. hirsutum* samples grouped by global breeding region (Mantel *r* = 0.257). Each dot represents a pairwise comparison between samples. Three small clusters below the main cluster of improved samples are circled in both figures with values based on SSR markers causing the separations. Each cluster is formed entirely by pairs of individuals with the common parent as labelled. The common parents are admixed samples as seen in Additional Figure A4.

a).


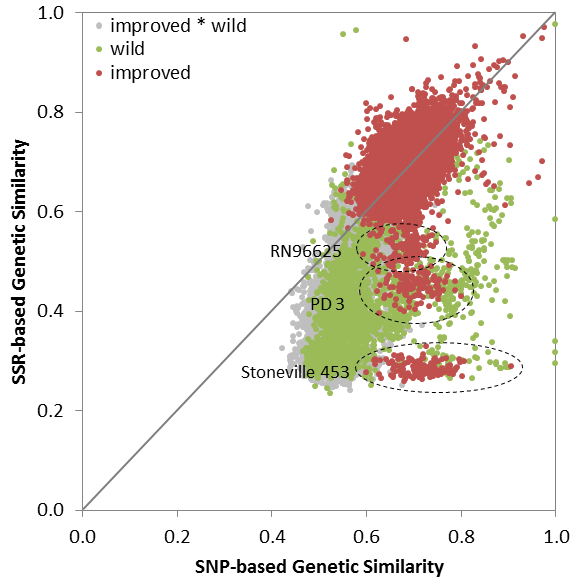


b).


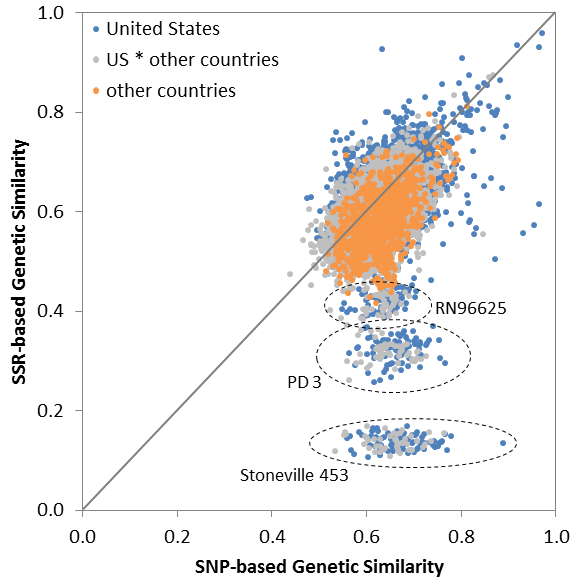


**References**

1. Lopez-Lavalle LAB, Gillespie VJ, Tate WA, Ellis MH, Stiller WN, Llewellyn DL, Wilson IW. Molecular mapping of a new source of Fusarium wilt resistance in tetraploid cotton (*Gossypium hirsutum* L.). Molecular Breeding 2012; 30:1181-91.

2. Stiller WN, Wilson IW. Australian cotton germplasm resources. In: World Cotton Germplasm Resources. Edited by Abdurakhmonov IY: InTech; 2014: 320.

3. Hinze L, Fang D, Gore M, Scheffler B, Yu J, Frelichowski J, Percy R. Molecular characterization of the *Gossypium* Diversity Reference Set of the US National Cotton Germplasm Collection. Theoretical and Applied Genetics 2015; 128:313-27.

4. Hinze LL, Gazave E, Gore MA, Fang DD, Scheffler BE, Yu JZ, Jones DC, Frelichowski J, Percy RG. Genetic diversity of the two commercial tetraploid cotton species in the *Gossypium* Diversity Reference Set. Journal of Heredity 2016; 107:274-86.

5. Grover C, Zhu X, Grupp K, Jareczek J, Gallagher J, Szadkowski E, Seijo J, Wendel J. Molecular confirmation of species status for the allopolyploid cotton species, *Gossypium ekmanianum* Wittmack. Genetic Resources and Crop Evolution 2015; 62:103-14.
